# Valley-dimensionality locking of superconductivity in cubic phosphides

**DOI:** 10.1126/sciadv.adf6758

**Published:** 2023-09-08

**Authors:** Lingyi Ao, Junwei Huang, Feng Qin, Zeya Li, Toshiya Ideue, Keivan Akhtari, Peng Chen, Xiangyu Bi, Caiyu Qiu, Dajian Huang, Long Chen, Rodion V. Belosludov, Huiyang Gou, Wencai Ren, Tsutomu Nojima, Yoshihiro Iwasa, Mohammad Saeed Bahramy, Hongtao Yuan

**Affiliations:** ^1^ National Laboratory of Solid State Microstructures, Jiangsu Key Laboratory of Artificial Functional Materials, College of Engineering and Applied Sciences, and Collaborative Innovation Center of Advanced Microstructures, Nanjing University, Nanjing 210000, China.; ^2^ Quantum-Phase Electronic Center and Department of Applied Physics, The University of Tokyo, Tokyo 113-8656, Japan.; ^3^ Institute for Solid State Physics, The University of Tokyo, Chiba 277-8581, Japan.; ^4^ Department of Physics, University of Kurdistan, Sanandaj 416, Iran.; ^5^ Center for High Pressure Science and Technology Advanced Research, Beijing 100094, China.; ^6^ Shenyang National Laboratory for Materials Science, Institute of Metal Research, Chinese Academy of Sciences, Shenyang 110016, China.; ^7^ Institute for Materials Research, Tohoku University, Sendai 980-8577, Japan.; ^8^ RIKEN Center for Emergent Matter Science, Wako, Saitama 351-0198, Japan.; ^9^ Department of Physics and Astronomy, School of Natural Sciences, The University of Manchester, Oxford Road, Manchester M13 9PL, UK.

## Abstract

Two-dimensional superconductivity is primarily realized in atomically thin layers through extreme exfoliation, epitaxial growth, or interfacial gating. Apart from their technical challenges, these approaches lack sufficient control over the Fermiology of superconducting systems. Here, we offer a Fermiology-engineering approach, allowing us to desirably tune the coherence length of Cooper pairs and the dimensionality of superconducting states in arsenic phosphides As*_x_*P_1−*x*_ under hydrostatic pressure. We demonstrate how this turns these compounds into tunable two-dimensional superconductors with a dome-shaped phase diagram even in the bulk limit. This peculiar behavior is shown to result from an unconventional valley-dimensionality locking mechanism, driven by a delicate competition between three-dimensional hole-type and two-dimensional electron-type energy pockets spatially separated in momentum space. The resulting dimensionality crossover is further discussed to be systematically controllable by pressure and stoichiometry tuning. Our findings pave a unique way to realize and control superconducting phases with special pairing and dimensional orders.

## Teaser

Valley-dimensionality–locked superconductivity is achieved via orbital selection in cubic AsP alloy under pressure.

## INTRODUCTION

Fermiology describes the electronic configuration of band structures and the shape of Fermi surfaces in superconducting systems. It plays important roles in determining the microscopic electron pairing mechanism and generating exotic quantum phenomena such as nematic states (*1*–*3*), Ising pairing (*4*–*7*), and spin-triplet pairing (*8*–*10*) as well as topological superconductivity (*11*). Normally, the coherence length ξ, serving as a key parameter of Fermiology, defines the spatial extension of the superconducting wave function and determines the dimensionality of superconductivity (*12*–*14*). Note that the practical limitation of the short coherence length of layered superconductors usually makes the realization of two-dimensional (2D) superconductivity technically challenging. Consequently, the achievement of 2D superconductivity strongly relies on reducing the superconducting thickness to the atomically thin limit in quantum confined systems (*6*, *15*, *16*) including cleaved atomically thin layers (*5*, *17*–*20*), epitaxially grown thin films (*21*–*26*), and gated interfaces of quantum materials (*27*–*30*). Alternatively, the 2D superconducting phase can, in principle, be realized by enlarging the coherence length via Fermiology engineering; however, in practice, this remains elusive.

**Fig. 1.**
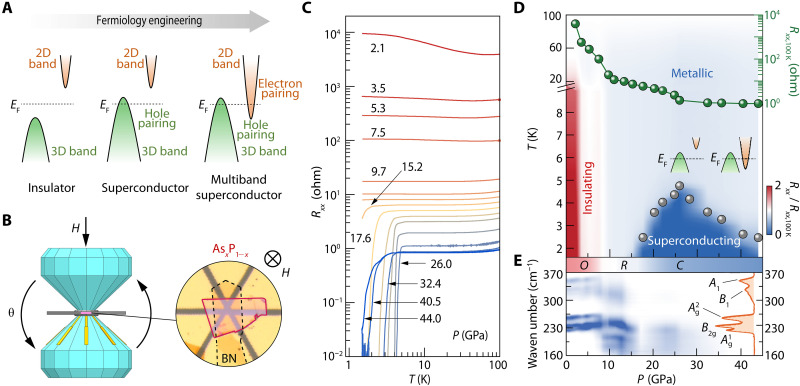
Pressure-induced energy-valley evolution and superconductivity in As*_x_*P_1−*x*_ nanoflakes. (**A**) Schematic illustration of valley-dimensionality–locked superconductivity tuned by Fermiology engineering. (**B**) Schematic view of diamond-anvil-cell (DAC) setup and microscopic photograph of As*_x_*P_1−*x*_ nanoflake. Left: Cartoon of DAC. Angle-dependent *H*_c2_ measurements were performed using a specially designed high-resolution DAC rotator, in which θ is the rotation angle. Right: Optical image of As*_x_*P_1−*x*_ nanoflake for transport measurements, in which the nanoflake (red edges) is encapsulated by hBN nanoflake (gray dashed edges). (**C**) Pressure-dependent *R_xx_*(*T*) curves of sample B (*x* = 0.65). (**D**) The superconducting phase diagram of sample B with colored mapping of normalized resistance *R_xx_*/*R*_*xx*,100 K_. Gray spheres represent the superconducting critical temperature *T*_c_, defined as the temperature with 50% resistance drops of its normal-state resistance at 10 K. The pressure-dependent *R*_*xx*,100 K_ is shown by green spheres. Insets schematically depict the evolution of electron- and hole-type valleys in the band structure. (**E**) Color mapping of pressure evolution of Raman spectra for sample F (*x* = 0.36). Raman spectrum at ambient pressure is shown with respect to the right axis. Italic letters *O*, *R*, and *C* indicate the orthorhombic, rhombohedral, and cubic crystal structural phases.

Here, we demonstrate a Fermiology engineering strategy using coherence length modulation to realize a dimensionality crossover of superconductivity from 3D to 2D in As*_x_*P_1−*x*_, combining diamond-anvil-cell high-pressure transport measurements and density functional theory (DFT) calculations. The 2D superconductivity is verified in bulk-like thick As*_x_*P_1−*x*_ nanoflakes (over 60 nm) by temperature- and angle-dependent upper critical field measurements together with Berezinskii-Kosterlitz-Thouless analysis. Theoretical calculations reveal that the observed dimensionality crossover arises from the generation of a new electron Fermi pocket at the vertices of the Brillouin zone (BZ), R points . Specifically, such a 3D-to-2D crossover result from the competition between two types of valleys in the band structure: smaller arsenic-derived electron pockets at the R point showing a longer out-of-plane coherence length and larger phosphide-derived hole pockets at the BZ center, Γ point, showing a shorter coherence length. Thus, these R- and Γ-valleys generate distinct 2D and 3D superconductivity correspondingly, leading to emergent valley-dimensionality locking of the superconductivity therein (Fig. 1A). Such valley-dimensionality–locked superconductivity is found to be mechanically tunable with pressure and chemically controllable with stoichiometry. This Fermiology engineering technique can generally be extended to other phosphide compounds, providing a practical route to achieving 2D superconductivity and shedding light on the valley-dimensionality locking mechanism of multiband superconductors.

## RESULTS AND DISCUSSION

We chose the van der Waals semiconductor As*_x_*P_1−*x*_ as the target material for the following two reasons. First, the reported value of the out-of-plane upper critical field is as small as 0.07 T (*31*) and directly suggests a correspondingly large ξ value (more than 60 nm based on our estimation), providing us with an ideal candidate to demonstrate coherence length engineering for realizing 2D superconductivity. Second, although As*_x_*P_1−*x*_ is a van der Waals band insulator with a layered crystal structure similar to black phosphorus (space group of *Cmca*), it turns to an isotropic cubic structure (space group of *Pm*$\bar{3}$*m*) under high pressure (*32*–*34*), in which the electronic properties and emergent superconductivity should be expected to behave with a 3D nature on the basis of the intuitive understanding that hydrostatic pressure normally drives superconducting systems with more 3D feature. Therefore, once we can observe 2D superconductivity in such an isotropic cubic system, it will greatly deepen our understanding of the electron pairing and dimensional orders of superconductivity.

To figure out the superconducting phase diagram of pressurized As*_x_*P_1−*x*_, we performed electronic transport measurements on As*_x_*P_1−*x*_ samples with an intentionally chosen thickness of 50 to 60 nm (samples A to E, figs. S1 to S3), which is much thicker than the atomically thin 2D limit of 0.6 nm (schematically shown in Fig. 1B). Taking sample B (*x* = 0.65) as an example, one can clearly see an insulator-metal-superconductor phase transition from the temperature-dependent resistance *R_xx_*(*T*) with increasing pressure (Fig. 1C). Specifically, a resistance drop appears in the *R_xx_*(*T*) curve at approximately 15.2 GPa at low temperature, indicating the onset of the superconducting transition, and the zero-resistance superconducting state is attained at 17.6 GPa. The superconducting critical temperature *T*_c_ first increases and then decreases with increasing pressure, showing dome-shaped behavior.

A typical electronic phase diagram of such pressure-induced superconductivity is given in Fig. 1D with a colored mapping of the normalized resistance (*R_xx_*/*R*_*xx*,100 K_) as a function of pressure and temperature (sample B, *x* = 0.65). The insulating, metallic, and superconducting phases can be clearly recognized, with the insulator-metal and metal-superconductor transitions occurring at 9.7 and 17.6 GPa, respectively. Note that two kinks in the pressure-dependent *R*_*xx*,100 K_ curve can be observed at 9.7 GPa (where the bandgap closes) and the pressure corresponding to maximum *T*_c_ (~26.0 GPa where new electron pockets appear, as shown in the insets of Fig. 1D). Because the superconductivity could be influenced by the scattering between the carriers (*35*, *36*), which can suppress the coherent behavior of emergent long-range order, the newly formed electron-type carriers might cause additional scattering (interband scattering) with decreasing *T*_c_ at pressures over 26.0 GPa, particularly between those electrons and holes. In particular, such pressure-modulated valleys in the band structure and resulting newly appeared electron pockets can be proven by quantitative analysis of 2D superconducting components (discussed later in the analysis of the upper critical magnetic fields).

**Fig. 2.**
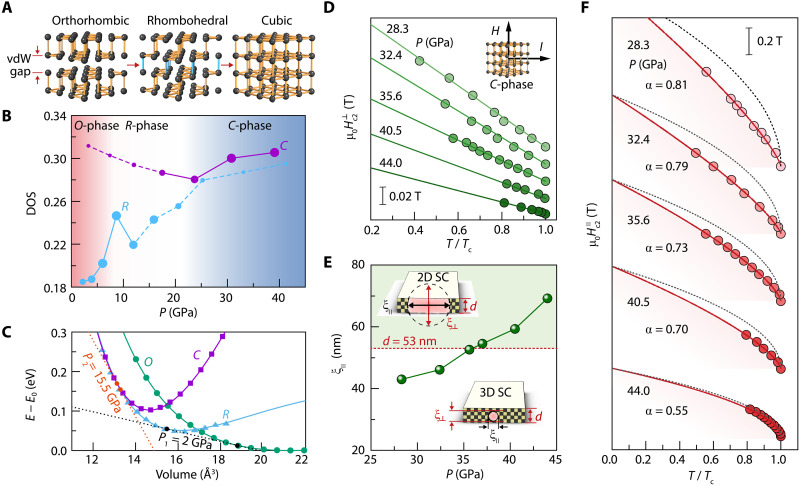
Structural phase transition and pressure dependence of *H*_c2_ properties in As*_x_*P_1−*x*_. (**A**) Structural evolution of As*_x_*P_1−*x*_ (*x* = 0) under pressure. vdW, van der Waals. (**B**) Pressure-dependent DOS for *R*-phase and *C*-phase structures at *x* = 0. Different sizes of circles and solid/dashed lines schematically indicate the proportion of *R*-/*C*-phase structures at the corresponding pressure. (**C**) Volume evolution of energy for three structures at *x* = 0. The slopes of the common tangents correspond to the critical pressures for *O-R* phase transition at *P*_1_ = 2.0 GPa and *R-C* phase transition at *P*_2_ = 15.5 GPa. (**D**) Temperature-dependent $H_{c2}^{⊥}$ of sample B at various pressures. *H*_c2_ is defined at 50% of the normal-state resistance drop. Solid curves are the fitted results by $H_{c2}^{⊥}(T)=H_{c2}^{⊥}(0)\left(1-\frac{T}{T_{c}}\right)$. (**E**) Pressure dependence of ξ_∥_ at 0 K for sample B. The values of ξ_∥_ evaluated on the basis of $H_{c2}^{⊥}(0)=\frac{{\text{Φ}}_{0}}{2\pi \xi_{∥}{\left(0\right)}^{2}}$ exceed the sample thickness of 53 nm (highlighted by the red dashed line). The mosaic squares in the lower inset schematically represent an isotropic structure with a small ξ_⊥_ at lower pressure. Increasing pressure can enhance ξ values to a level larger than the sample thickness *d* (shown in the upper inset). SC, superconductivity. (**F**) Temperature-dependent $H_{c2}^{∥}$ of sample B. The black dashed curves are fitted results by $H_{c2}^{∥}$ ∝ (1 − *T*/*T*_c_)^0.5^. Red curves are fitted with $H_{c2}^{∥}$ ∝ (1 − *T*/*T*_c_)^α^.

The corresponding critical pressures in the electronic phase diagram are associated with the structural phase transitions (*31*, *32*), in which As*_x_*P_1−*x*_ turns from an orthorhombic structure (*O*-phase) to a rhombohedral structure (*R*-phase) and lastly reaches a cubic structure (*C*-phase) under high pressure (figs. S4 and S5). The superconductivity appears after the *O*-*R* structural transition, confirmed by the pressure evolution of Raman spectra with five active vibration modes (Fig. 1E). These structural phase transitions in the As*_x_*P_1−*x*_ compound, schematically shown in Fig. 2A, can be further theoretically verified with the kink behaviors of the pressure-dependent electronic density of state at the Fermi level for the *R*- and *C*-phases (Fig. 2B) and with the corresponding volume-dependent free energies for the *O*-, *R*- and *C*-structural phases under pressure (Fig. 2C). In this study, we will mainly focus on the emergent superconductivity in As*_x_*P_1−*x*_ with a high-pressure cubic structure. Given that the *C*-phase is structurally isotropic, the electronic properties and the ξ of superconductivity should also become isotropic therein, in sharp contrast with the anisotropic electronic features of the layered *O*-phase. As will be discussed below, this enables us to develop an unusual strategy through Fermiology engineering to control the dimensionality and pairing orders in a superconducting system.

To evaluate the superconducting coherence length of the superconducting state in cubic As*_x_*P_1−*x*_, we performed temperature-dependent measurements of the upper critical magnetic field *H*_c2_(*T*) (defined as the magnetic field where the resistance drops to 50% of its normal-state value) based on the Ginzburg-Landau analysis (*12*). As shown in Fig. 2D (sample B), the temperature dependence of the out-of-plane upper critical field, $H_{c2}^{⊥}(T)$ can be well fitted by the following 3D Ginzburg-Landau formula: $H_{c2}^{⊥}(T)=H_{c2}^{⊥}(0)\left(1-\frac{T}{T_{c}}\right)$ and $H_{c2}^{⊥}(0)=\frac{{\text{Φ}}_{0}}{2\pi \xi_{∥}{\left(0\right)}^{2}}$, where Φ_0_ is the magnetic flux quantum and ξ_∥_(0) is the zero-temperature value of the superconducting coherence length. Because the fitted $H_{c2}^{⊥}(0)$ values monotonically decrease with increasing pressure, the resulting ξ_∥_(0) values monotonically increase without reaching a saturated value (Fig. 2E and figs. S6 and S7). Two things need to be addressed here: (i) The ξ_∥_(0) value is enhanced markedly with increasing pressure and even becomes larger than the sample thickness *d* of 53 nm. (ii) On the basis of our x-ray diffraction (XRD) results and DFT calculations, the As*_x_*P_1−*x*_ sample is lastly pressurized into an isotropic cubic structure above ~30 GPa, implying that the out-of-plane coherence length ξ_⊥_ will become equal to the in-plane coherence length ξ_∥_. Note that the anisotropy of upper critical magnetic fields can be obtained on the basis of the shape anisotropy of the disk-like sample, especially where ξ_⊥_ is similar to *d* (more details in section S5). The combination of the above two aspects provides us with the opportunity to achieve the expected dimensionality crossover of the superconducting state from 3D to 2D in these compressed bulk-like samples (inset of Fig. 2E).

To understand the pressure-induced evolution of the dimensionality of the superconducting state, we measured the in-plane upper critical field $H_{c2}^{∥}(T)$ at different pressures on sample B (Fig. 2F). The red solid lines are the fitting curves for data at various pressures based on the relation $H_{c2}^{∥}$ ∝ (1 − *T*/*T*_c_)^α^, where α is the crucial parameter to phenomenologically reflect the dimensionality evolution of superconductivity: α = 1 is for 3D superconductivity at the limit of *d*/ξ_⊥_ → ∞, α = 0.5 is for 2D superconductivity at the limit of *d*/ξ_⊥_ → 0, and 1 > α > 0.5 can reflect the intermediate states of superconductivity with finite *d*/ξ_⊥_ values during the dimensionality crossover. With increasing pressure, the fitted α value gradually approaches 0.5, indicating that the dimensionality of the superconductivity gradually approaches the 2D nature (more details in figs. S8 to S11), as verified by the 2D Ginzburg-Landau formula (*12*): $H_{c2}^{∥}(T)=\frac{\sqrt{12}{\text{Φ}}_{0}}{2\pi \xi_{∥}(0)d_{\mathrm{SC}}}{\left(1-\frac{T}{T_{c}}\right)}^{0.5}$, where *d*_SC_ denotes the effective superconducting thickness. We find ξ_∥_(0) = 69.2 nm and *d*_SC_ = 35.1 nm for sample B at 44.0 GPa. Because the thickness of the sample decreases with increasing pressure, the value of *d*_SC_ is less than the sample thickness measured at ambient conditions via the atomic force microscopy (AFM) measurement. Such a good fit based on the 2D Ginzburg-Landau model clearly demonstrates the 2D nature of superconductivity in sample B at high pressure. Thus, the dimensionality of the superconductivity in As*_x_*P_1−*x*_ (*x* = 0.65) can be tuned from 3D to 2D by increasing the applied pressure.

A comparison between the coherence length and the sample thickness *d* can help us better understand and recognize such a dimensionality crossover in superconductivity. Because As*_x_*P_1−*x*_ shows a structural phase transition from a strongly anisotropic layered structure to an isotropic cubic structure (*31*, *32*), the ξ_⊥_ values monotonically increase and eventually reach a value close to ξ_∥_, which provides us with the possibility of achieving the isotropic coherence length of superconductivity under high pressure. We thus believe that the criterion of *d* < ξ_∥_ can be specifically used for this isotropic cubic phase to confirm the 2D nature of the high-pressure–induced superconductivity in the structurally isotropic superconductor. For example, as discussed above, the ξ_∥_ values of sample B reach a value of approximately 70 nm at 44.0 GPa, greatly exceeding the sample thickness of 53 nm (Fig. 2E and fig. S1). This sample at 44.0 GPa shows 2D superconductivity according to the criterion of *d* < ξ_∥_. On the basis of the high-pressure cubic structure and the superconductivity therein, we believe that the ξ_⊥_ values are greatly enhanced by applying pressure and become identical to the ξ_∥_ values exceeding the sample thickness, thus resulting in an isotropic coherence length of superconductivity.

Note that the superconductivity in cubic As*_x_*P_1−*x*_ is isotropic in coherence length and dominated by the orbital limit (fig. S12 and table S1; the value of *H*_c2_ is remarkably lower than the Pauli paramagnetic limiting field), which is distinct from the recently observed isotropic superconductivity reaching the Pauli limit in infinite-layer nickelate (*37*). Unlike the pressure-induced 2D-to-3D dimensionality crossover of superconductivity reported in bismuth-based cuprate (*38*), our observed 3D-to-2D crossover with different evolution directions, in particular the achieved 2D superconductivity under high pressure, is unexpectedly contrary to the expectation that hydrostatic high pressure normally drives the dimensionality of superconductivity into 3D. This dimensionality crossover of superconductivity can be understood by the marked enhancement of the ξ_⊥_ values, demonstrating that Fermiology engineering can be a powerful tool for achieving 2D superconductivity. Only in such a case with a certain sample thickness, we can observe the superconductivity dimensionality crossover with the appearance of the electron pocket at the Fermi surface tuned by physical pressure and chemical stoichiometry (figs. S13 to S15). Therefore, the dimensionality is locked to the different carrier pockets at the Fermi surface.

As mentioned above, there is a pressure-induced carrier-type transition from hole to electron (sign change of Hall coefficient *R*_H_, figs. S16 and S17) at the pressure showing maximum *T*_c_ of superconductivity, which implies the band structure evolution and corresponding modulation on the Fermiology by applying pressure. Then, we checked the stoichiometry-dependent Fermiology evolution and resulting superconducting properties of the pressurized As*_x_*P_1−*x*_ samples. On the one hand, when increasing the stoichiometry *x*, the critical pressure of the metal-superconductor transition moves toward a higher value, and the maximum *T*_c_ decreases correspondingly (figs. S18 and S19 and table S2). According to our DFT calculations, because As*_x_*P_1−*x*_ is a Bardeen-Cooper-Schrieffer (BCS) superconductor formed by phonon-mediated electron pairing (*39*), the underlying mechanism of the decrease in maximum *T*_c_ with changing *x* is similar to the well-known isotope effect of superconductivity (*12*), in which the phonon energies of As*_x_*P_1−*x*_ decrease with heavier arsenic element doped into the cubic phosphorus lattice (fig. S3).

**Fig. 3.**
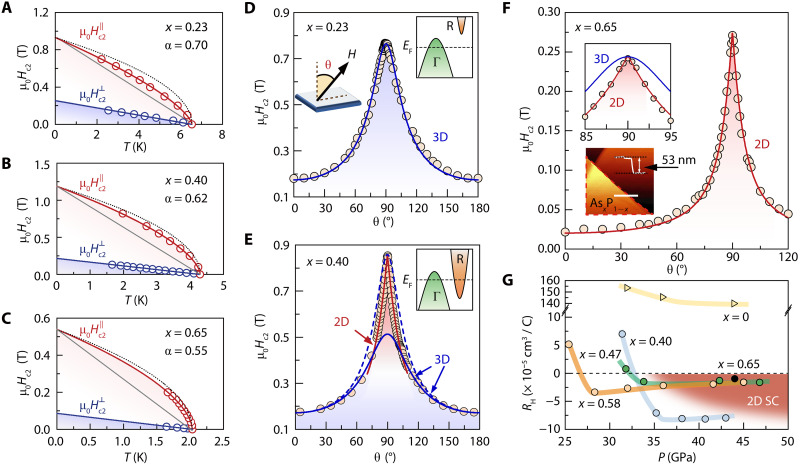
Superconducting dimensionality depending on the stoichiometry in cubic As*_x_*P_1−*x*_. (**A** to **C**) Temperature dependence of both $H_{c2}^{∥}$ and $H_{c2}^{⊥}$ for sample A (*x* = 0.23) at 43.1 GPa, sample C (*x* = 0.40) at 43.0 GPa, and sample B (*x* = 0.65) at 44.0 GPa. Black dashed curves are fitted lines with $H_{c2}^{∥}$ ∝ (1 − *T*/*T*_c_)^0.5^. The blue and gray curves are fitted lines with *H*_c2_ ∝ 1 − *T*/*T*_c_. The red solid curves are fitted lines with $H_{c2}^{∥}$ ∝ (1 − *T*/*T*_c_)^α^. (**D** to **F**) Angle-dependent *H*_c2_ at 1.5 K for samples A, C, and B. The orange circles represent the experimental data points. The blue and red curves represent the fitting results from the3D Ginzburg-Landau model and 2D Tinkham model, respectively. Right insets of (D) and (E) schematically show hole- and electron-type valleys for different stoichiometries. Lower inset of (F) shows that the thickness of sample B is 53 nm. (**G**) Pressure-dependent *R*_H_ derived by *R*_H_ ∝ *R_xy_d*∕*H*. *R_xy_* is the Hall resistance and *d* is the sample thickness. The data for *x* = 0 in (G) are adopted from reference *34*. The data for *x =* 0.40 and *x* = 0.65 were obtained at 1.6 K, and the data for *x =* 0.47 and *x* = 0.58 were obtained at 10 K.

On the other hand, at a pressure of 43.0 GPa, the superconductivity will be driven into a 2D state with increasing stoichiometry *x*. Such a phenomenon originates from a systematic change in the ionicity of neighboring elements in cubic As*_x_*P_1−*x*_, causing a drastic spatial redistribution of the wave function of the conducting electrons. Figure 3 (A to C) shows the temperature-dependent $H_{c2}^{⊥}$ and *H*_c2_^∥^ at a similar pressure of approximately 43.0 GPa for three samples with different stoichiometries *x*. With increasing stoichiometry *x*, the $H_{c2}^{∥}(T)$, data gradually approach the square-root relation. Specifically, the $H_{c2}^{∥}(T)$ data of As*_x_*P_1−*x*_ with lower *x* are closer to the 3D Ginzburg-Landau model (Fig. 3A), while those of As*_x_*P_1−*x*_ with higher *x* can be well fitted to the 2D Ginzburg-Landau model (Fig. 3C). As discussed later, the extended nature of the As wave function can profoundly modify the Fermiology and the dimensional order of the superconducting states in pressurized As*_x_*P_1−*x*_ samples.

To provide additional evidence for 2D superconductivity beyond the above *H*_c2_(*T*) analysis, we performed angle-dependent upper critical field *H*_c2_ measurements *H*_c2_(θ) using a specially designed high-resolution diamond-anvil-cell rotator (θ is the angle between the applied magnetic field and the normal direction of the sample surface, as shown in the inset of Fig. 3D). On the basis of the Ginzburg-Landau theory (*12*), the expressions of *H*_c2_(θ) for the anisotropic 3D superconductor and the 2D superconductor are given as follows: ${\left[\frac{H_{c2}(\theta )\cos\theta }{H_{c2}^{⊥}}\right]}^{2}+{\left[\frac{H_{c2}(\theta )\sin\theta }{H_{c2}^{∥}}\right]}^{2}=1$ and $|\frac{H_{c2}(\theta )\cos\theta }{H_{c2}^{⊥}}|+{\left[\frac{H_{c2}(\theta )\sin\theta }{H_{c2}^{∥}}\right]}^{2}=1$, where $H_{c2}^{⊥}$ and $H_{c2}^{∥}$ correspond to the upper critical magnetic fields at θ = 0° and θ = 90°, respectively. Specifically, one can see a clear evolution of the superconductivity dimensionality from 3D, to coexisting 3D and 2D, and lastly to 2D with increasing stoichiometry *x* (Fig. 3, D to F, and figs. S20 to S22).

For sample A at 43.1 GPa (*x* = 0.23, Fig. 3D), the *H*_c2_(θ) curve exhibits a round-shaped peak near θ = 90° and can be well fitted by the 3D Ginzburg-Landau model over the whole angle range, indicating 3D superconductivity therein. For sample C at 43.0 GPa (*x* = 0.40, Fig. 3E), the *H*_c2_(θ) curve exhibits a cusp-shaped peak and can be fitted by the 2D Tinkham model in the range of θ = 90 ± 10° (diverging from the 3D anisotropic Ginzburg-Landau model, marked by blue dashed line) and can be fitted by the 3D Ginzburg-Landau model for the remaining θ ranges (blue solid line). Note that only by combining 2D and 3D pairing models based on piecewise function analysis in different angle ranges can the *H*_c2_(θ) data be fitted well, and the 2D component becomes acutely pronounced at approximately θ = 90°. In sharp contrast to the mixed behavior, the *H*_c2_(θ) data of sample B at 44.0 GPa (*x* = 0.65, Fig. 3F) can be well fitted by the 2D Tinkham model rather than the 3D Ginzburg-Landau model for the whole θ region, as emphasized in the enlarged inset. Such analyses of superconducting dimensionality from the *H*_c2_(θ) data are in good accordance with the analyses based on the *H*_c2_(*T*) data in Fig. 3 (A to C). Note that 2D (3D) superconductivity, as mentioned above, still includes negligible 3D (2D) superconducting components, which can be confirmed by the quantitative analysis of superconducting dimensionality components (fig. S22).

The radical response of *H*_c2_(θ) and the dimensionality crossover of superconductivity to external pressure and As doping suggest a drastic change in the Fermiology of the conducting states. The high-pressure cubic structure allows the outer *p_x_*, *p_y_*, and *p_z_* orbitals of P/As to form σ-type covalent bonds with their first-nearest neighbors along the *x*, *y*, and *z* axes, respectively. Accordingly, one can expect that the resulting Fermi surfaces from these orbitals are hole-type, forming around the BZ center with a threefold degeneracy at the Γ point (inset of Fig. 3D). Therefore, the superconductivity arising from hole pairing within these orbitals exhibits a 3D-like behavior. Doping arsenic, which has more extended *p* orbitals than phosphorus, can trigger extra bonding between the next-nearest and even second-nearest neighbors due to short ionic distances at such high pressure. This accordingly leads to additional electron pockets elsewhere at the BZ boundaries with very different pairing orders, as shown in the inset of Fig. 3E (discussed later). Thus, both hole and electron pockets can be fully gapped by effectively hosting Cooper pairs in arsenic-rich samples at high pressure, resulting in an orbital-driven multigap BCS superconductor with both hole and electron carriers at different valleys.

Owing to the orthogonality between those orbitals forming the Fermi pockets in Fermiology, each superconducting component formed by electron/hole pairing within a specific valley is individual and weakly coupled to each other (*40*). However, the initial Γ-centered one has a shorter coherence length (due to its more considerable volume) than its counterparts at BZ boundaries (due to its smaller volume). Therefore, those pockets from two types of valleys behave in different dimensions; the Γ-centered one shows 3D behavior, while its counterparts at BZ boundaries show 2D behavior. Such a valley-dependent characteristic of superconductivity suggests that our samples can be effectively considered multiband superconductors in which the 3D hole-type Γ-valley and 2D electron-type R-valley coexist and determine the nature of the dominant pairing orders and their dimensionalities. This superconducting feature can be directly confirmed by Hall effect measurements, as shown in Fig. 3G. The sign of the Hall coefficient *R*_H_ changes from positive to negative, and the effective electron density gradually increases with increasing *x* and pressure, reflecting the band structure evolution and Fermiology modulation by changing chemical stoichiometry and physical pressure (figs. S13 to S17).

**Fig. 4.**
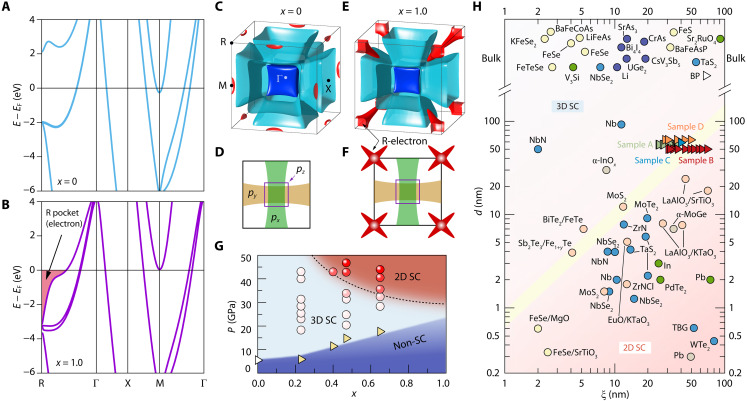
Evidence of the dimensionality crossover of valley-dimensionality–locked superconductivity in cubic As*_x_*P_1−*x*_. (**A** and **B**) Electronic band structure of As*_x_*P_1−*x*_ for *x* = 0 and *x* = 1 at 50 GPa. (**C** to **F**) Fermi surface and the corresponding projection onto the *k_x_*-*k_y_* plane at 50 GPa for *x* = 0 in (C) and (D) and for *x* = 1 in (E) and (F). An extra electron-type valley crossing the Fermi level around the BZ vertices (R point) forms relatively small star-shaped electron pockets, contributing to the valley-selective 2D superconductivity. (**G**) Phase diagram of superconductivity dimensionality as a function of pressure and stoichiometry *x* of As*_x_*P_1−*x*_. The triangle defines the boundary between non-superconducting (non-SC) region and superconductivity (SC) region, with a white triangle adopted from (*31*) and yellow triangles derived from our transport results. The color of spheres from white to red indicates the dimensionality of superconductivity from 3D to 2D, which is obtained by the angle-dependent *H*_c2_ results. (**H**) Survey of superconducting materials in comparison of ξ with *d*. Green, red, blue, and orange triangles represent data taken from samples A, B, C, and D. The yellow line represents *d* = ξ. The data points are adopted from those references listed in table S3.

To clarify this superconducting feature and its different dimensionalities, we calculated the electronic band structures of the end compounds *x* = 0 (Fig. 4A) and *x* = 1 (Fig. 4B) at 50 GPa in the cubic phase. For *x* = 0, i.e., cubic phosphorus, there is effectively only one set of hole-type valleys centered at the Γ point crossing the Fermi level. Because of their *p*-orbital character, as explained above, they form large axially oriented Fermi surfaces along all three Γ-X directions in the BZ (see Fig. 4, C and D). In the *x* = 1 compound, i.e., cubic arsenic, in addition to these hole-type valleys, we can also see an electron-type valley crossing the Fermi level around the BZ vertices, R point, forming a relatively small star-shaped Fermi surface (see Fig. 4, E and F). These two carrier-type valleys are dominant in different superconducting dimensionalities. In the superconducting phase, we thus expect that the coexistence between the hole-type Γ-valleys and electron-type R-valleys can lead to the observed crossover from 3D to 2D superconductivity.

Figure 4G summarizes the phase diagram of the superconducting dimensionality evolution as a function of pressure and stoichiometry in As*_x_*P_1−*x*_. Three distinct regions can be clearly identified upon increasing pressure: the non-superconducting region (insulator and normal metal state, shaded in royal blue), the 3D superconductivity region (shaded in light blue), and the 2D superconductivity region (shaded in red). Specifically, the critical pressures for the metal-superconductor transition (denoted as triangles) of different samples with various *x* are obtained from the corresponding pressure-dependent *R_xx_*(*T*) curves. The boundary between 3D and 2D superconductivity is established from the analyses of *H*_c2_(θ) under different pressures, particularly for cubic As*_x_*P_1−*x*_ for higher arsenic doping. On the basis of such a systematic phase diagram, we thus believe that Fermiology engineering by tuning high pressure and stoichiometry can serve as a feasible way to realize intrinsic 2D superconductivity even in thick superconducting samples with thicknesses above 60 nm. Note that these bulk-like thick samples show typical 2D superconducting characteristics of the Berezinskii-Kosterlitz-Thouless transition (detailed analysis shown in figs. S23 and S24).

Figure 4H provides a comprehensive summary of the *d*-ξ diagram with superconducting dimensional orders as a function of ξ and *d* of reported superconducting materials (table S3). For our samples, the ξ values gradually increase and go across the *d* = ξ boundary (yellow line) at high pressure, systematically enabling a crossover from 3D to 2D superconductivity. Two important features of the *d*-ξ diagram need to be addressed here. On the one hand, in contrast to 2D superconductors with ultrathin superconducting thicknesses (*6*, *15*, *16*, *41*) located below the critical line *d* = ξ, we have successfully realized 2D superconductivity with a superconducting thickness above 60 nm, which is seldomly feasible in those traditional cases without achieving an atomically thin limit. On the other hand, with pressure-driven coherence length engineering, we can remarkably enhance the coherence length in our isotropic superconductor to 70 nm under 44 GPa, which is comparable to those well-known cases of ultralong coherence lengths in oxide heterostructures (*22*, *42*, *43*). Unlike the 2D superconductivity achieved by reducing the sample thickness to the atomically thin limit (vertically crossing the critical line *d* = ξ from top to bottom in the *d*-ξ diagram of Fig. 4H), our results present an alternative strategy for 2D superconductivity by achieving valley-selective superconductivity and thereby enhancing ξ in thick samples with a clean high-pressure technique (horizontally crossing the critical line in the *d*-ξ diagram).

In summary, we demonstrated a special approach to realizing valley-dimensionality locking of superconductivity through a delicate but profound manipulation of the Fermiology of the energy states in momentum space. Unlike the conventional approach to achieving 2D superconductivity by reducing the thickness of the superconducting layer to an atomically thin limit, the mechanism described in this work enables the realization of 2D superconductivity with distinct Fermiological features in bulk-like samples. The observed superconductivity in As*_x_*P_1−*x*_ emerges from the quantum states formed by electron and hole carriers with distinct dimensional orders. Having access to such a disparity provides a unique opportunity to study a broad range of quantum criticality phenomena, such as insulator-to-superconductor phase transition, competition between long-range electronic orders, quantum Lifshitz phase transition of topology change in Fermiology, the appearance of non-Fermi liquid behavior, and universality with scaling behavior and critical exponents. We have noticed that Fermiology engineering can be more generally applied to other phosphide compounds via multiple control of mechanical and stoichiometric methods. The insights obtained from this work also shed light on the physics of collective quantum phenomena emerging from the interplay between internal degrees of freedom of electrons subject to external perturbations.

## MATERIALS AND METHODS

### Growth of van der Waals As*_x_*P_1−*x*_ single crystals

Single crystals of As*_x_*P_1−*x*_ were prepared by the gas-phase transformation method (*44*). Here, red phosphorus and arsenic powders with a certain stoichiometric ratio were used as raw materials, and all the other parameters remained the same as those in the preparation of black phosphorus. Specifically, there are four steps to obtain the As*_x_*P_1−*x*_ crystal. (i) The raw materials, AuSn alloy and SnI_4_, were sealed in a quartz ampoule with a length of 13 cm and a diameter of 56 cm, and the pressure of the quartz ampoule was lower than 10^−3^ mbar. (ii) The sealed ampoule was placed horizontally in a tubular furnace (Lindberg Blue M) and heated to 650°C within 1 hour. (iii) After being kept at this temperature for 24 hours, the ampoule was cooled to 500°C at a rate of 40°C per hour. On the basis of the above process, a large-size As*_x_*P_1−*x*_ crystal can form in the cold end of the ampoule. (iv) The As*_x_*P_1−*x*_ bulk crystals were eventually collected and washed with hot toluene, deionized water, and acetone in sequence. NOTE: Special attention should be given to the use of arsenic.

### Electronic transport measurements in diamond anvil cell

The high-pressure technique with a screw-type diamond anvil cell made of nonmagnetic beryllium-copper alloy was used for electronic transport measurements. Diamond anvils with 300-μm-diameter culets can produce a high pressure up to 50 GPa. A predrilled hole with a diameter of 260 μm at the preindented center area (40 μm) of a T301 stainless steel gasket (thickness of 250 μm) was used as the sample chamber. The metal gasket was isolated from the electrodes by pressing mixed powders of cubic boron nitride with epoxy. As*_x_*P_1−*x*_ nanoflakes were mechanically exfoliated from bulk crystals with polydimethylsiloxane and then transferred onto the diamond culet with prepatterned Ti/Au electrodes (thickness of 4/20 nm) by a dry-transfer process. The nanoflakes were then capped by a thin hBN nanoflake for protection. The thickness of the As*_x_*P_1−*x*_ nanoflakes cleaved on top of the diamond surface (fig. S1) was precisely identified by AFM (WITec Alpha 300). Electronic transport measurements were performed in a cryofree integrated superconducting magnet system (TeslatronPT, Oxford Instruments) with the lowest temperature down to 1.5 K and a maximum magnetic field up to 14 T. Four-terminal resistance was obtained by lock-in amplifiers (SR830, Stanford Research Systems) with a measurement frequency of 13 Hz. The angle-dependent transport measurements were performed by using a specially designed high-resolution diamond-anvil-cell rotator with an angular resolution better than 0.1°, which provides us with a unique technical platform with additional freedom to precisely control the sample orientation relative to the direction of the magnetic field and systematically study the pressure-induced superconductivity under different magnetic field directions.

### High-pressure Raman and XRD measurements

Raman and photoluminescence spectra were obtained by using a confocal Raman microscopy system (WITec Alpha 300) with an excitation laser of 532 nm, a grating of 1800 mm^−1^, and a ×50 Olympus objective lens to achieve a small spot size of less than 1 μm^2^. Raman spectra at room temperature were collected with an integration time of 20 s and accumulated five times. In situ high-pressure powder XRD was performed using the powder mode of a Bruker D8 Venture diffractometer with Mo Kα radiation (λ = 0.7107 Å) in a diamond anvil cell with 300-μm-diameter culets. The powder sample was placed into a 150-μm-diameter hole drilled in a Re gasket prepressed to 40-μm thickness. No pressure-transmitting medium was used to avoid sample contamination. The precise value of the applied pressure was calibrated by using the photoluminescence of ruby microballs in the sample chamber at room temperature (*45*).

### First-principles calculations

Electronic structure calculations were performed within the DFT using Perdew-Burke-Ernzerhof exchange-correlation (*46*) functionals and ultrasoft pseudopotentials as implemented in the VASP program (*47*, *48*). The crystal structure and atomic positions of elemental P and As were fully optimized in all three phases—orthorhombic (*O*), rhombohedral (*R*), and cubic (*C*) structures—for a range of volumes *V* until the magnitude of the force on each ion became less than 0.001 eV/Å. The total cutoff energy was set to 500 eV. The corresponding BZs of the *O*-, *R*,- and *C*-phases were sampled by 13 × 13 × 13, 20 × 20 × 20, and 25 × 25 × 25 *k*-meshes, respectively. All relativistic effects, including spin-orbit coupling, were fully taken into account. The resulting total energy *E*(*V*) for each phase was fitted using the third-order isothermal Birch-Murnaghan equation of state (*49*–*51*)

$$E(V)=E_{0}+\frac{9V_{0}B_{0}}{16}\left\{{\left[{\left(\frac{V_{0}}{V}\right)}^{\frac{2}{3}}-1\right]}^{3}B_{0}^{\prime }+{\left[{\left(\frac{V_{0}}{V}\right)}^{\frac{2}{3}}-1\right]}^{2}\left[6-4{\left(\frac{V_{0}}{V}\right)}^{\frac{2}{3}}\right]\right\}$$

where *E*_0_ is the free energy at zero pressure, *V*_0_ is the reference volume, *B*_0_ is the bulk modulus, and $B_{0}^{\prime }$ is the first derivative of the bulk modulus with respect to pressure. The critical phase transitions were obtained by finding the common tangents between the relevant *E*(*V*) curves deduced from the above equation.

## Supplementary Materials

### This PDF file includes:

Supplementary Text Figs. S1 to S24 Tables S1 to S3 References
