# Supplementary material for: Valley-dimensionality locking of superconductivity in cubic phosphides
Source: Sci Adv. 2023 Sep 8;9(36):eadf6758. doi: 10.1126/sciadv.adf6758 (PMC10491139; doi:10.1126/sciadv.adf6758)
Supplement: Supplementary file 1 — Supplementary Text Figs. S1 to S24 Tables S1 to S3 References [file sciadv.adf6758_sm.pdf]

Science Advances
AAAS

Supplementary Materials for

Valley-dimensionality locking of superconductivity in cubic phosphides

Lingyi Ao *et al.*

Corresponding author: Hongtao Yuan, htyuan@nju.edu.cn; Mohammad Saeed Bahramy, m.saeed.bahramy@manchester.ac.uk; Yoshihiro Iwasa, iwasa@ap.t.u-tokyo.ac.jp

Sci. Adv. **9**, eadf6758 (2023)
DOI: 10.1126/sciadv.adf6758

This PDF file includes:

Supplementary Text
Figs. S1 to S24
Tables S1 to S3
References

Supplementary Text

1. Transferring As_xP_{1-x} nanoflakes with the desired thickness on the diamond culet of DAC

To prepare the device with the desired thickness for transport measurements, we mechanically transferred As_xP_{1-x} nanoflakes onto a diamond culet of DAC setup to achieve 2D superconductivity by coherence length engineering. The thickness values of those As_xP_{1-x} nanoflakes are intentionally selected in the range of 50–60 nm and accurately identified by AFM measurement. Figure S1 shows the corresponding optical microscopic images (left panel), AFM images (middle panel) and the cross-sectional profiles of AFM topography (right panel) for each As_xP_{1-x} sample. The cross-sectional profiles are taken along the red lines as indicated in the AFM images, where the thicknesses of samples A, B, C, D and E are confirmed to be 56 nm, 53 nm, 59 nm, 63 nm and 41 nm, respectively. One can see that these nanoflakes are atomically flat, ensuring uniform thickness in each of the whole samples for high-pressure transport measurement. Therefore, we can have an excellent material platform to explore 2D superconductivity by coherence length engineering.

2. Determination of the x value of As_xP_{1-x} and anisotropic evolution under high pressure

To quantitatively understand the influence of the stoichiometry x on the Fermiology and resulting superconductivity in As_xP_{1-x}, we established a practical method to build up a correspondence of the x-dependent Raman spectrum to the precise x level obtained from energy-dispersive X-ray spectroscopy (EDS). Examples of element composition mapping images of P and As elements in As_xP_{1-x} nanoflakes are shown in Fig. S2. Note that the P and As elements are uniformly distributed throughout the entire nanoflake in a homogeneous way. Figure S3 shows our Raman results of As_xP_{1-x} samples with very different stoichiometry x in the range of 0 to 0.6, based on which we can establish the specific relation between Raman peak positions and the stoichiometry x of the As_xP_{1-x} samples determined from the EDS measurements. One can see that the Raman spectrum of $x = 0$ (pure black phosphorus) presents three obvious vibration peaks, which correspond to the A_g^1 (360 cm^{-1}), B_{2g} (437 cm^{-1}) and A_g^2 (464 cm^{-1}) phonon modes, in accordance with a previous report (*52*). With increasing As doping level, five new Raman peaks appear in the Raman spectrum of As_xP_{1-x} samples (x = 0.2, 0.4, 0.6), contributed by the appearance of As–P bonds and As–As bonds in their crystal structures. More specifically, there are three groups of Raman vibration modes that are independently contributed from the P–P bonds (Fig. S3B), the As–P bonds (Fig. S3C) and the As–As bonds (Fig. S3D) inside the As_xP_{1-x} crystal. It is clear that the vibration frequencies of those modes decrease sharply as the x value increases. In particular, five vibration modes, including As-A_g^1, As-B_{2g}, A_1, B_1, P-B_{2g}, and P-A_g^2, show systematic redshift behavior (gray shade), which can be used to confirm the x value of our exfoliated samples inside DAC. For example, with this optical method, we quickly identified the x value of sample A (shown in the main text) to be 0.23, which reasonably matches the value confirmed by EDS measurements.

To gain better insight into the evolution of anisotropy in Raman spectra under pressure, we obtained room-temperature polarization-dependent Raman spectra of an As_xP_{1-x} sample (x = 0.36) at different pressures, as shown in Fig. S4. Two points need to be addressed here. On the one hand, the evolution of Raman peak positions with pressure clearly corresponds to the above-mentioned structural transitions from the *O*-phase to the *R*-phase and finally to the *C*-phase. As shown in Fig. S4A, a new Raman peak appears at 218 cm^{-1} with a pressure increase of 8.2 GPa, corresponding to the formation of the *R*-phase. This peak shows a slight redshift within the *R*-phase with increased pressure, while it starts to show a fast redshift once the *C*-phase appears at a

pressure above 17.6 GPa. On the other hand, there is anisotropy in the Raman spectra. At ambient pressure, the polarization dependence of the Raman peak at 240 cm^{-1} shows fourfold rotational symmetry, confirming that the pristine *O*-phase of As_xP_{1-x} is structurally anisotropic. As shown in Fig. S4, B to G, the polarization dependence gradually evolves from fourfold at the pressure corresponding to the *O*-phase (Fig. S4E at 2.0 GPa) to twofold at the pressure corresponding to the *R*-phase (Fig. S4F at 10.3 GPa) and finally to one-fold at the pressure corresponding to the *C*-phase (Fig. S4G at 46.6 GPa), indicating that the anisotropy feature of the As_xP_{1-x} crystal changes with increasing pressure. This is consistent with theoretical calculations for the structural phase evolution of the As_xP_{1-x} crystal under pressure.

3. High-pressure XRD measurements of As_xP_{1-x}

To clarify the crystal structure of As_xP_{1-x} under high pressure, we performed high-pressure XRD measurements of As_xP_{1-x}. The As_xP_{1-x} crystal at ambient pressure shows an orthorhombic structure (*54–56*) identical to its parent material of black phosphorus. Specifically, the pressure evolution of the XRD spectra of As_xP_{1-x} (x = 0.4) is shown in Fig. S5, A and B. One can see that As_xP_{1-x} undergoes two structural phase transitions from the *O*-phase to the *R*-phase and finally to the *C*-phase at high pressure, consistent with a previous report (*33*). To clearly address the structural evolution of As_xP_{1-x}, we extracted the pressure evolution of the main Bragg peak positions ranging from 14° to 26°, as shown in Fig. S5C. The Bragg peaks of (220) (gray balls) and the Bragg peaks of (230) (green balls) disappear at approximately 7 GPa, representing the appearance of the *R*-phase. The *O*-phase still partially exists until the pressure reaches approximately 11 GPa, where the peak of (122) (black balls) disappears and a new peak (pink balls) appears, implying that As_xP_{1-x} undergoes a phase transition to the *R*-phase. With further increasing pressure, two peaks (pink and red balls) gradually merge into a new peak (blue balls), corresponding to a partial transformation of the *R*-phase to the *C*-phase. At a pressure above 23 GPa, As_xP_{1-x} can be stable in the pure *C*-phase, where the Bragg peak of (111) (light blue balls) disappears. This phenomenon of two structural transitions is in good accordance with the XRD spectrum of black phosphorus (*34*). Note that after decompression, as shown in Fig. S5D, all the XRD peaks return to their original positions before compression, indicating that such structural phase transitions are reversible.

4. Coherence length analysis from the upper critical magnetic field

To understand the superconducting dimensional orders of As_xP_{1-x} samples under pressure, we carried out temperature-dependent measurements of the upper critical magnetic field H_{c2} and performed Ginzburg–Landau analysis. With such analysis for the temperature-dependent resistance and magnetic field-dependent resistance near the superconducting transition (Fig. S6), we can obtain the values of $H_{c2}^{\perp}(0)$ and the in-plane coherence length at zero temperature $\xi_{\parallel}(0)$ to quantitatively understand the pressure evolution of $\xi_{\parallel}(0)$ with different chemical stoichiometries.

One can see in Fig. S7 that the $H_{c2}(T)$ values of sample A can be well fitted by the Ginzburg–Landau formula in the main text. The obtained $H_{c2}^{\perp}(0)$ values gradually decrease with increasing pressure from 10.4 GPa to 28.4 GPa and then start to saturate with further increased pressure (Fig. S7C), resulting in saturation of the coherence length $\xi_{\parallel}(0)$ at pressures higher than 28.4 GPa (Fig. S7D). In contrast, with a higher x value in sample B, the $H_{c2}^{\perp}(0)$ values monotonically decrease with increasing pressure without reaching saturation (Fig. S7C), resulting in a dramatic

enhancement of the $\xi_{\|}(0)$ values larger than its thickness (53 nm). Such results, together with an isotropic cubic structure under high pressure (implying the isotropic coherence length of superconductivity therein, $\xi_{\|} \approx \xi_{\perp}$), provide the possibility for the realization of dimensionality crossover of superconductivity in compressed As_xP_{1-x} with a higher x value.

5. The anisotropy of upper critical magnetic fields of cubic As_xP_{1-x} with a certain thickness

The shape anisotropy in the superconductivity can be well understood by Ginzburg–Landau theory, in which the values of the upper critical fields are associated with the boundary condition of the superconductor. Normally, when the superconductivity is under an external magnetic field, the free energy F of an isotropic superconductor near the transition temperature T_c can be expressed in terms of an order parameter φ, which is the pseudo wave function related to the density of the superconducting component and only nonzero below T_c.

$$F = F_0 + \int \left(\alpha|\varphi|^2 + \frac{\beta}{2}|\varphi|^4 + \frac{1}{2m}|\vec{p}\varphi|^2 + \frac{\mu_0 \vec{H}^2}{2} \right) d\vec{r}^3$$

where $\vec{p} = -i\hbar\vec{\nabla} - 2e\vec{A}$ is the momentum operator and $\vec{A}$ is the vector potential of magnetic field $\vec{H}$ satisfying $\vec{\nabla} \times \vec{A} = \vec{H}$.

To minimize this free energy by applying the variation of the order parameter φ

$$\frac{\delta}{\delta\varphi^*} F = 0$$

Thus, we obtain the GL equation

$$\alpha\varphi + \beta|\varphi|^2\varphi + \frac{\vec{p}^2}{2m}\varphi = 0$$

For the state near phase transition ($|\varphi|^2 \sim 0$), an equality of which the solution indicates the critical line in the phase diagram.

$$\left(\alpha + \frac{\vec{p}^2}{2m}\right)\varphi = 0$$

It is clear that when applying the magnetic field, the kinetic term cannot be neglected since the cooper pairs are in the cyclotron motion, which is nothing but the vortex state.

According to the Landau level, such cyclotron motion contributes kinetic energy as

$$\frac{\vec{p}^2}{2m} = \left(n + \frac{1}{2}\right)\hbar\omega_c$$

where $n = 0, 1, 2, \ldots$ and $\omega_c = 2e\mu_0 H/m$ is the Larmor frequency for charge $2e$.

Thus,

$$\alpha + \frac{\vec{p}^2}{2m} = -\alpha_0\left(1 - \frac{T}{T_c}\right) + \left(n + \frac{1}{2}\right)\hbar\omega_c \leq 0$$

which gives the upper critical field

$$\mu_0 H \leq \frac{m}{\left(n + \frac{1}{2}\right)2\hbar e}\alpha_0\left(1 - \frac{T}{T_c}\right) \leq \frac{m\alpha_0}{\hbar e}\left(1 - \frac{T}{T_c}\right) = \frac{2m\alpha_0}{\hbar^2}\frac{\hbar}{2e}\left(1 - \frac{T}{T_c}\right) = \frac{\Phi_0}{2\pi\xi(0)^2}\left(1 - \frac{T}{T_c}\right)$$

where $\xi(0)^2 = \frac{\hbar^2}{2m\alpha_0}$ is the coherent length at zero temperature and $\Phi_0 = h/2e$ is the magnetic flux quantum.

Such an upper critical field is the orbital limit. Each vortex has a unit magnetic flux quantum of Φ_0 and a finite size of $\pi\xi^2$, in which the inner state is not superconducting. When the magnetic

field increases, the density of vortices increases, the non-superconducting region grows, and thus, the upper critical field is given when vortices touch each other, which is nothing but

$$\mu_0 H_{c2} = \frac{\Phi_0}{2\pi\xi(0)^2}\left(1 - \frac{T}{T_c}\right) \propto \frac{\Phi_0}{\pi\xi^2}$$

Note that for the thin superconductor, when the external magnetic field is applied along the in-plane direction, the upper critical field $\mu_0 H_{c2}$ displays a linear dependence on the temperature, showing the 3D nature of the superconductivity where the Cooper pairs are not restricted by any boundary condition of the superconductor. This corresponds to the case of ξ being much smaller than d.

Importantly, for a superconductor with a finite size, particularly when ξ is similar to d, the motion of the Cooper pairs is strongly restricted by the boundary of the superconductor. This affects the cyclotron motion of the Cooper pairs and causes compression of the vortex state, thus resulting in modification of the Landau levels therein.

In an extreme case when ξ is much larger than d to solve the GL equation, this leads to the Tinkham solution of the upper critical field, which is nothing but

$$\mu_0 H_{c2} = \frac{\sqrt{12}\Phi_0}{2\pi d\xi(0)}\left(1 - \frac{T}{T_c}\right) \propto \frac{\Phi_0}{\pi d\xi}$$

Based on this solution, the in-plane upper critical field is enhanced by a factor of ξ/d (ξ is much larger than d); thus, the superconductivity displays extreme anisotropy in the upper critical field between the in-plane and out-of-plane directions.

Therefore, one can conclude that for a superconductor with a finite size, since the motion of the Cooper pairs is strongly restricted and thus the Landau levels are modified, the superconductivity will display an anisotropy in the upper critical field between the in-plane and out-of-plane directions. This is an intuitive result of the shape anisotropy in the thin superconductor, even though the exact solution when ξ is similar to d is analytically difficult to obtain.

6. The origin of 2D superconductivity in cubic As_xP_{1-x}

There are two important length parameters to determine the superconductivity dimensionality: the sample thickness d and the superconducting coherence length $\xi_\perp$ along the out-of-plane direction. When $d \gg \xi_\perp$, the superconductivity is three-dimensional. In sharp contrast, when $d \ll \xi_\perp$, the superconductivity is two-dimensional. Importantly, in the superconductivity dimensionality crossover regime when $d \sim \xi_\perp$, the superconductivity shows intermediate dimensionality behavior. Since we only focus on the superconductivity dimensional crossover in this manuscript, samples are intentionally chosen with thickness d near 50 nm, which are on a similar length scale to the superconducting coherence length $\xi_\perp$ along the out-of-plane direction. In our AsP system, there are two types of carriers, and the effective superconductivity dimensionality is thus hybridized with the superconductivity dimensionality of each type of carrier. Specifically, hole-type and electron-type carriers can have different superconducting coherence lengths due to their different Fermiologies, enabling them to display distinct dimensional orders in superconductivity in a sample depending on its thickness. The hole-type pocket due to its *p*-orbital character has a large axially oriented Fermi surface in the extended along Γ-X directions in the Brillouin zone (BZ). This allows the corresponding Cooper pairs to gain relatively large wave vectors and hence an overall short superconducting coherence length

ξ_h. As such, they can exhibit a 3D-type superconductivity even in relatively thin samples. In sharp contrast, the electron-type pocket emerging under high pressure at the BZ vertices, R points, are much smaller and less dispersive than their hole counterparts, thereby enabling a relatively longer superconducting coherence length ξ_e. As such, when the sample thickness d satisfies $\xi_e > d > \xi_h$, the electron and hole carriers exhibit distinct superconductivity dimensionality behaviors: hole-type carriers will show 3D-like superconducting behavior, and electron-type carriers will show 2D-like superconducting behavior. This accordingly implies the dimensionality is locked to the different carrier pockets at the Fermi surface.

More importantly, as illustrated in Fig. S8, our calculations reveal a monotonic growth of the R pocket with increasing pressure. This suggests that the effective density of states of the R pocket, and hence its contribution to superconductivity, is enhanced under pressure. This behavior is consistent with our expectation that a dimensional crossover from 3D to 2D superconductivity occurs at sufficiently high x values and pressures, due to the 2D-type nature of superconductivity arising from the R pocket.

Based on the above discussion, although the hole-type carrier (3D superconducting component) always exists, the effective superconducting dimensionality can be different from 3D and thus evolves to 2D when the newly formed electron-type carrier is dominant in the superconducting pairing. Note that only in the case that our sample has a certain thickness on a length scale near 50 nm can we observe such superconductivity dimensionality crossover, together with the appearance of the electron pocket at the Fermi surface, tuned by chemical stoichiometry and physical pressure. As shown in Fig. S9, we obtained pressure-dependent α fitting parameters of sample A and sample B based on $H_{c2}^{\parallel} \propto (1 - T/T_c)^{\alpha}$. One can see that the value of α for sample A remains about 0.7 with increasing pressure because the coherence length values are not larger than its thickness. In contrast, with a higher x value in sample B, the α values gradually decrease to 0.5 with increasing pressure (Fig. S9C), and the coherence length values gradually increase to larger than its thickness (Fig. S7C), indicating that the dimensionality of the superconductivity in As_xP_{1-x} (x = 0.65) can be tuned from 3D to 2D by increasing the applied pressure.

7. Analysis of the upper critical magnetic field by 2D Ginzburg–Landau theory

The analysis of the pressure-induced evolution of the dimensionality of the superconducting state in the main text is based on $H_{c2}^{\parallel} \propto (1 - T/T_c)^{\alpha}$. The fitting parameter α value can phenomenologically reflect the dimensionality evolution of superconductivity, in which α = 1 is for 3D superconductivity, α = 0.5 is for 2D superconductivity, and 1 > α > 0.5 can reflect the intermediate states of superconductivity during dimensionality crossover. To clearly reflect the 2D behavior at high pressure and the occurrence of dimensionality crossover from 3D to 2D with applied pressure, we fitted $\mu_0 H_{c2}^{\parallel}$(T) data by $(1 - T/T_c)^{0.5}$ based on the 2D Ginzburg–Landau model. The results are shown in Fig. S10. One can see that our $\mu_0 H_{c2}^{\parallel}$(T) data are more consistent with the 2D Ginzburg–Landau model under high pressure, and the mean squared error (MSE) of the optimal fitting curve can also prove this, as shown in Fig. S10F. The MSE → 0 limit and the value of α → 0.5 indicate that the dimensionality of the superconductivity gradually approaches a 2D nature. Therefore, dimensionality crossover from 3D to 2D in As_xP_{1-x} can be observed regardless of which model is used for fitting.

More interestingly, the anisotropy ratio $H_{c2}^{\parallel}/H_{c2}^{\perp}$ as a function of T/T_c with increasing pressure and stoichiometry x is shown in Fig. S11. Specifically, the curves $H_{c2}^{\parallel}/H_{c2}^{\perp} \propto (1 - T/T_c)^{\alpha-1}$ at different α ($1 \geq \alpha \geq 0.5$) are given to reflect the intermediate states of superconductivity during the dimensionality crossover from 3D to 2D (Fig. S11A). As shown in Fig. S11B, with increasing pressure, the anisotropy ratio $H_{c2}^{\parallel}/H_{c2}^{\perp}$ gradually approaches $(1 - T/T_c)^{-0.5}$, indicating that the dimensionality of the superconductivity approaches a 2D nature. Similarly, the anisotropy ratio $H_{c2}^{\parallel}/H_{c2}^{\perp}$ gradually approaches $(1 - T/T_c)^{-0.5}$ with increasing x (Fig. S11C). Such observations that the curve of the $H_{c2}^{\parallel}/H_{c2}^{\perp}$ value as a function of T/T_c approaches $(1 - T/T_c)^{-0.5}$ (characterized by 2D superconductivity) clearly indicate the superconductivity dimensionality crossover from 3D to 2D with increasing pressure or x.
Note that the upper critical magnetic field of the superconductivity in our AsP system is dominated by the orbital limit for the following two reasons. First, the $H_{c2}^{\parallel}(0)$ value is far less than the Pauli limit $\mu_0 H_{c2}^{\mathrm{Pauli}}$ value. This implies that the vanishing of superconductivity under magnetic field is not due to the break-down of spin-singlet Cooper pairs from the Pauli paramagnetic effect in the weak coupling BCS theory. Second, the temperature-dependent upper critical magnetic field $H_{c2}(T)$ is indeed consistent with the Werthamer-Herfand-Hohenberg (WHH) model (*57*). Taking sample A ($x = 0.23$) as an example, we estimated the value of the orbital limit $\mu_0 H_{c2}^{\mathrm{orb}}(0)$ at different pressures (Fig. S12) by a linear fitting near T_c (the resulting slope $\left.\frac{dH_{c2}}{dT}\right|_{T=T_c}$ and other relevant specific parameters are shown in Table S1). One can see that, at each pressure, the value of $H_{c2}^{\perp}$ at 0 K approaches the corresponding value of $\mu_0 H_{c2}^{\mathrm{orb}}(0)$ and the Maki parameter $\alpha_M \ll 1$, indicating that the depairing mechanism indeed dominates the orbital effect (at which the current density around vortices reaches a pair breaking value) and that the Pauli paramagnetic effect therein is negligible.

8. Dimensionality crossover and corresponding Fermiology modulation of the superconducting state in cubic As_xP_{1-x} at high pressure

The dimensionality crossover from 3D to 2D superconductivity is confirmed with the emerging electron pocket in Fermiology, as presented in Fig. 4 in the main text. To further determine the valley–dimensionality locking nature of superconductivity and the corresponding Fermiology modulation under pressure, we performed angle-dependent and temperature-dependent upper critical field H_{c2} experiments to determine the superconductivity dimensionality. More importantly, to establish a better connection between the Fermi surface change and 2D superconducting behavior, we systematically studied the corresponding pressure-dependent Hall coefficient R_H.
Taking sample D and sample E as examples, the $H_{c2}(T)$ relations for in-plane and out-of-plane magnetic field orientations under different pressures on sample D are shown in Fig. S13, A and B. The fitting parameter α (an indicator of dimensionality) based on $H_{c2}^{\parallel} \propto (1 - T/T_c)^{\alpha}$ clearly changes from ~0.65 (at 31.8 GPa) to ~0.5 (at 33.8 GPa) with increased pressure, indicating the evolution from 3D to 2D superconductivity with increased pressure, similar to sample B (Fig. 2E). For the experimental data at 33.8 GPa, where $\alpha \approx 0.5$, we determined the superconducting thickness d_{SC} and found that the fitted value of d_{SC} (approximately 57 nm) is comparable to the sample thickness d (63 nm at ambient pressure), confirming the 2D superconductivity under high

pressure. More importantly, such an evolution of superconducting dimensionality can be further confirmed by the $H_{c2}(\theta)$ data in a direct way, as shown in Fig. S13, C and D. The $H_{c2}(\theta)$ at 31.8 GPa can be well fitted by the 3D Ginzburg–Landau model, exhibiting a round-shaped peak near $\theta = 90°$, while the round-shaped behavior of $H_{c2}(\theta)$ becomes less pronounced at higher pressure and approaches the cusp shape (2D Tinkham model). To confirm that such a dimensionality evolution of superconductivity originates from the appearance of extra electron pockets of cubic As_xP_{1-x}, we performed Hall effect measurements and determined the carrier density n_{3D} (Fig. S13, E and F). One can see that the slope of the $R_{xy}(\mu_0 H^{\perp})$ relation varies from positive to negative at a particular pressure when 2D superconductivity starts to appear (33.8 GPa, Fig. S13F), indicating that the major carrier type varies from hole to electron.

Similarly, the Hall data for sample E ($x = 0.58$, 41-nm-thick) at different pressures and the corresponding analysis of the temperature-dependent upper critical magnetic fields are shown in Fig. S14. Three points need to be addressed here. First, the slope of the $R_{xy}(H)$ relation changes from positive to negative with increasing pressure (Fig. S14 A and B), indicating that the sign of R_H for sample E changes from positive to negative. Second, the $|R_H|$ value decreases with increasing pressure, implying that the effective electron density gradually increases. Third, to further confirm that the 2D superconductivity results from the newly-appeared electron pockets, we obtained pressure-dependent fitted α values for sample E (Fig. S14 C and D) based on the same analysis model $H_{c2}^{\parallel} \propto (1 - T/T_c)^{\alpha}$ as that used in Fig. 2F. One can see that the fitted α value gradually approaches 0.5 with increasing pressure, indicating that the dimensionality of the superconductivity gradually approaches 2D. Such an observation for sample E is consistent with the behaviors for sample B in Fig. 2F (pressure-induced 3D-to-2D dimensionality-crossover of superconductivity). Note that the Hall data for sample B at 44.0 GPa ($\alpha \approx 0.55$, Fig. 2F) also demonstrates a negative R_H and larger effective electron density (Fig. S15).

Therefore, those are in good accordance with the pressure-modulated valleys in the band structure and the emergence of electron pockets based on the DFT calculations in the main text. Such observations further confirm the valley–dimensionality locking of superconductivity in cubic As_xP_{1-x}.

9. Estimated pressure values for newly-appeared electron pockets of As_xP_{1-x}

Normally, the sign change of R_H from positive to negative is accompanied by the electron pocket dropping below the Fermi level. Thus, we can obtain the pressure value at which the electron pocket appears by pressure-dependent Hall measurements in a specific sample. Taking the stoichiometric value $x = 0.40$ as an example (Fig. S16A), we estimate that the sign of R_H changes from positive to negative at a pressure P_{h-e} of approximately 33 GPa (P_{h-e} denotes the pressure value at which the electron pocket drops below the Fermin level) based on the smooth trend guided by the eyes. Similarly, we can obtain the pressure values of P_{h-e} for different samples. For $x = 0.47$, P_{h-e} is approximately 32 GPa (Fig. S16B), and for $x = 0.58$, P_{h-e} is approximately 27 GPa (Fig. S16C). As a result, the relation between the stoichiometry value x and the pressure value at which the electron pocket appears is shown in Fig. S16D. Based on such a stoichiometry-dependent tendency in a linear approximation, we extrapolate the fitted line of stoichiometry-dependent P_{h-e} to the stoichiometry value $x = 0.65$ (sample B) and thus roughly estimate that the pressure value P_{h-e} of sample B is approximately 24 GPa, similar to the pressure 26 GPa at the maximum T_c value.

To establish a better connection between the Fermi surface change and the appearance of 2D superconducting behavior, we systematically studied the stoichiometry-dependent and pressure-dependent Hall coefficient R_{H} in As_xP_{1-x} (Fig. S17 and Fig. 3G in the main text). Three points need to be addressed as follows.
First, to clearly understand the stoichiometry-dependent evolution of the Fermi surface, we performed Hall effect measurements for samples with different x values at a fixed pressure of ~43 GPa. One can see that the sign of R_{H} values changes from positive to negative with increasing x (Fig. S17), indicating that the major carrier type varies from hole to electron. Combined with the analysis of the upper critical magnetic field, 2D superconductivity can be observed at higher x values at which an electron pocket emerges based on DFT calculations (Fig. 4A and B in the main text). Therefore, this can serve as the first piece of evidence to confirm the connection between the change in Fermi surface and the appearance of 2D superconductivity.
Second, to clearly understand the pressure-dependent evolution of the Fermi surface, we performed Hall measurements on samples C, D and E with increasing pressure (Fig. S16). Similarly, the sign of R_{H} changes from positive to negative with increasing pressure, implying that the major carrier type varies from hole to electron. The $|R_{\mathrm{H}}|$ value decreases with increasing pressure at a higher x value of $x = 0.58$, indicating that the effective electron density gradually increases. Combined with the analysis of the upper critical magnetic field, 2D superconductivity can be observed at higher pressure values at which an electron pocket emerges. Therefore, this can serve as the second piece of evidence to confirm the connection between the change in the Fermi surface and the appearance of 2D superconductivity.
Third, to build up the phase diagram between the Hall coefficient (reflecting the change in Fermi surface) and the dimensionality of superconductivity, we also replotted the phase diagram of superconductivity dimensionality as a function of pressure and Hall coefficient in Fig. 3G. One can clearly see that 2D superconductivity appears when the Hall coefficient is negative and its absolute value is relatively small. In such a case, the effective electron density is higher; thus, the observations further confirm that the appearance of 2D superconductivity results from newly-appeared electron pockets.

10. Stoichiometry-dependent superconducting phase diagram of As_xP_{1-x}

To further investigate the stoichiometry effect on the pressure-induced superconductivity in a series of As_xP_{1-x} samples, we performed temperature-dependent resistance measurements and summarized the pressure-dependent phase diagrams in Fig. S18 and Table S2. One can see that the P_{MS} for the metal-superconductor transition in As_xP_{1-x} systematically increases with increasing x value, while the corresponding $T_{\mathrm{c,Max}}$ (for the maximum T_{c} values) decreases. Similar dome-shaped superconducting behavior can be achieved in our samples, followed by the metal-superconductor transition with the application of pressure. To clearly observe this behavior, taking sample A ($x = 0.23$) and sample B ($x = 0.65$) as examples, we plotted the corresponding phase diagram, as shown in Fig. S19 and Fig. 1D of the main text, respectively.

11. Detailed $H_{\mathrm{c2}}(\theta)$ data for the determination of 2D superconductivity

The analysis of the dimensionality crossover from 3D to 2D superconductivity in the main text is based on the specific definitions of H_{c2} (the magnetic field at which R_{xx} becomes 50% of the

normal state resistance R_N). To show the robustness of such dimensionality crossover and the validity of such analysis, more generally, we used different H_{c2} definitions to analyze the angular dependence of the upper critical field H_{c2}. The fitting results with different definitions of $10\%R_N$, $30\%R_N$ and $50\%R_N$ are shown in Fig. S20. The $H_{c2}(\theta)$ data of sample D at 1.5 K and 46.8 GPa can be fitted well by the 2D Tinkham model (solid red line) rather than the 3D Ginzburg–Landau model (solid blue line), regardless of whether the value of $H_{c2}(\theta)$ is determined by $10\%R_N$ (Fig. S20A), $30\%R_N$ (Fig. S20C) or $50\%R_N$ (Fig. S20E), indicating the robustness of the 2D nature of superconductivity and the solid validity of our analysis method.
To prove that the dimensionality crossover of superconductivity can generally occur at other temperatures, we performed angular-dependent upper critical field measurements at temperatures below T_c. Figure S21 shows the $H_{c2}(\theta)$ data of sample D at different temperatures. One can see that the $H_{c2}(\theta)$ data (determined by $50\%R_N$) can be well fitted by the 2D Tinkham model rather than the 3D Ginzburg–Landau model. Such an observation clearly indicates that the achieved 2D superconductivity is robust to existing at other temperatures below T_c.
Furthermore, to better illustrate the superconductivity dimensionality crossover from 3D to 2D with increasing pressure and *x*, a hybrid model (*58*) can be used to show the pressure-dependent and stoichiometry-dependent dimensionality evolution: $\left[\frac{H_{c2}(\theta)\sin\theta}{H_{c2}^{\parallel}}\right]^2 = a\left[\frac{H_{c2}(\theta)\cos\theta}{H_{c2}^{\perp}}\right]^2 + b\left|\frac{H_{c2}(\theta)\cos\theta}{H_{c2}^{\perp}}\right| + 1$, where the fitting parameters $|a| + |b| = 1$, essentially quantify the relative contribution of $H_{c2}(\theta)$ from an anisotropic 3D superconductivity and a 2D superconductivity therein. Specifically, the value of $|b|$ is closer to 1 with increasing *x* and pressure (Fig. S22), which confirms our results that 2D superconductor-like behavior appears at higher *x* and pressure, consistent with the phase diagram of superconductivity dimensionality in Fig. 4G of the main text.

12. Berezinskii–Kosterlitz–Thouless phase transition for 2D superconductivity
To understand the vortex-antivortex pairing in the observed 2D superconductivity, we performed Berezinskii–Kosterlitz–Thouless (BKT) analysis on our low-temperature electronic transport results. Here, two typical transport characteristics are given for a BKT transition analysis.
The first method is based on the voltage-current $V(I)$ characteristics of $V \propto I^{\beta}$ (β is a power-law exponent) when the current is larger than the corresponding I_c (*22*, *59*). Figure S23A shows the $V(I)$ characteristics measured on sample D at 33.8 GPa from 1.6 K to 4.0 K. One can see that the fitting curves are linear (black line) on a logarithmic scale, indicating that the relation between V and I satisfies the power law relation. We can deduce that the temperature of the BKT transition ($T_{BKT,IV}$) is 2.48 K when β = 3, as shown in Fig. S23B.
The second method is based on the Halperin–Nelson equation (*60*, *61*):

$$R = R_0 \exp\left(-\gamma t^{-\frac{1}{2}}\right) \quad \text{(S1)}$$

where t is the reduced temperature of $\frac{T}{T_{BKT}} - 1$, and R_0 and γ are the fitting parameters depending on the material properties. From the $R_{xx}(T)$ curve with equation S1, we can deduce that the value of $T_{BKT,RT}$ is 2.46 K at 33.8 GPa (Fig. S23C). Note that the values of $T_{BKT,IV}$ and $T_{BKT,RT}$ are almost equal under different pressures, indicating the existence of the BKT transition in As_xP_{1-x}, where 2D superconductivity exists.
We further expanded the analysis involving the existence of the BKT transition to further determine the superfluid stiffness together with its temperature dependence and estimate the

Pearl length, as well as the penetration depth. On the one hand, we determined the superfluid stiffness (*62*) $J_S[K] = (\beta - 1)\frac{T}{\pi}$ and its temperature dependence based on $V \propto I^\beta$ in the $I - V$ curves at different temperatures. One can see that the $I - V$ curve evolves from linear in the normal state ($T > T_c$) to nonlinear in the superconducting state ($T < T_c$). Importantly, at the temperature of T_{BKT} = 2.48 K, the β value reaches 3, and $J_S(T_{BKT})$ = 1.58 K. Moreover, the value of superfluid stiffness J_S approaches zero above T_c = 2.83 K, as shown in Fig. S23D. On the other hand, we estimated Pearl screening length (*63*) $\Lambda = \frac{2\lambda^2}{d_{BKT}}$ = 7.85 mm of sample D at 33.8 GPa by equation (*62*), expressed as $J_S(T = T_{BKT})[K] = \frac{2}{\pi}T_{BKT} = 6.2\frac{d_{BKT}[nm]}{\lambda^2[\mu m^2]}$. Correspondingly, the penetration depth $\lambda(T_{BKT})$ was estimated to be 15 μm, considering the effective transverse length scale d_{BKT} ≈57.2 nm (the superconducting thickness).

We also check the following consistency in the BKT analysis, including i) the recovery of the linear $I - V$ behavior and ii) the abrupt jump at the critical current, as shown in Fig. S24. One can see that the linear $I - V$ behavior clearly recovered at 4 K, at which temperature the sample is in the normal state. As a result, the value of β reaches 1 (indicating linear $I - V$ behavior) when the temperature is far above T_c. Importantly, there is an abrupt jump from nonlinear to linear behavior at the critical current. Although the current here is not large enough to observe the linear behavior, the nonlinear region is more important, which needs to fix a small range in data acquisition to acquire small signals, and the larger current may damage the sample.

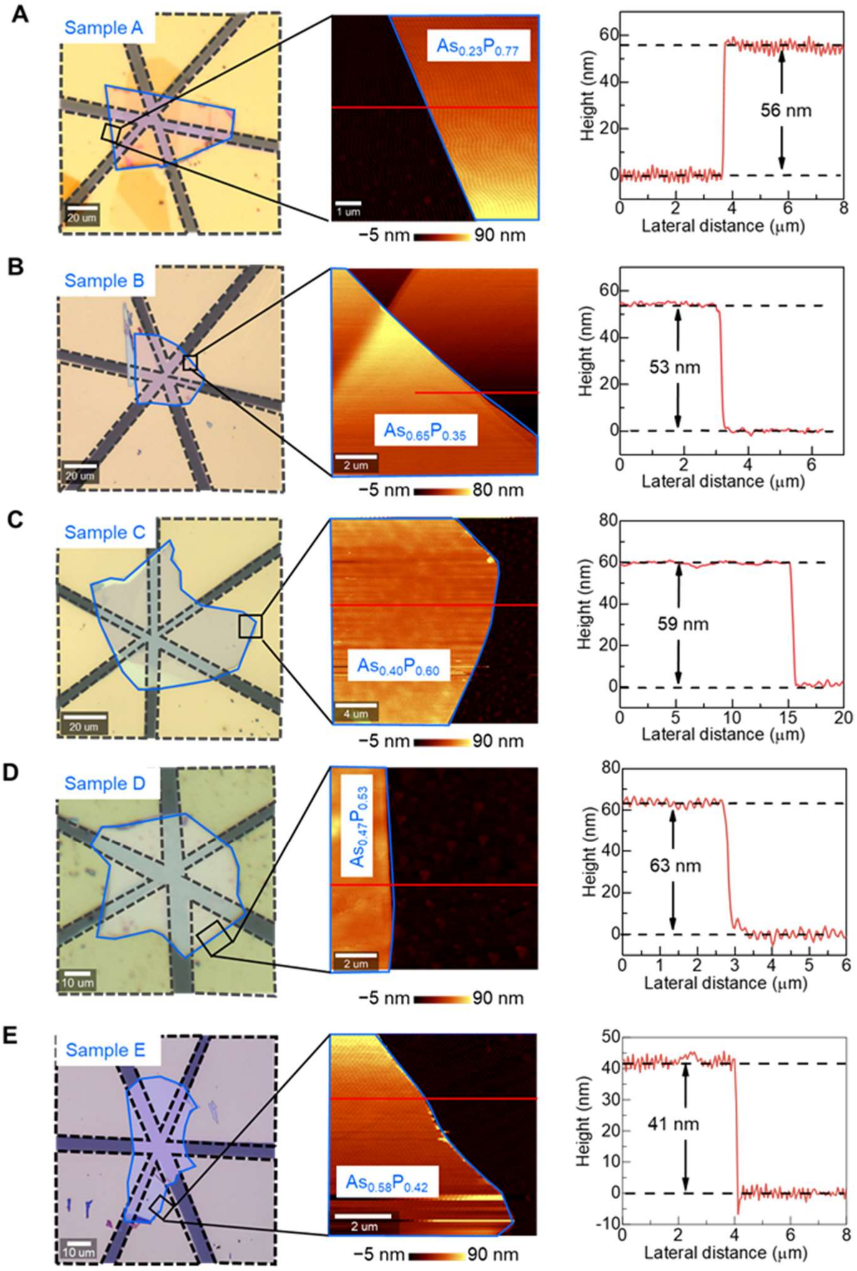

Fig. S1.

Experimental confirmation of the thickness of As_xP_{1-x} nanoflakes on the diamond surface of the DAC setup. (**A** to **E**) Optical images (left column), corresponding AFM images (middle column) and the cross-sectional profiles of AFM topography on the diamond surface (right column) for samples A, B, C, D and E. The sample (blue edges) is transferred onto the top of the diamond surface with prepatterned Ti/Au electrodes (black dashed). The cross-sectional profiles are taken along the red lines shown in AFM images, in which the thicknesses are estimated to be 56, 53, 59, 63 and 41 nm for samples A, B, C, D and E, respectively.

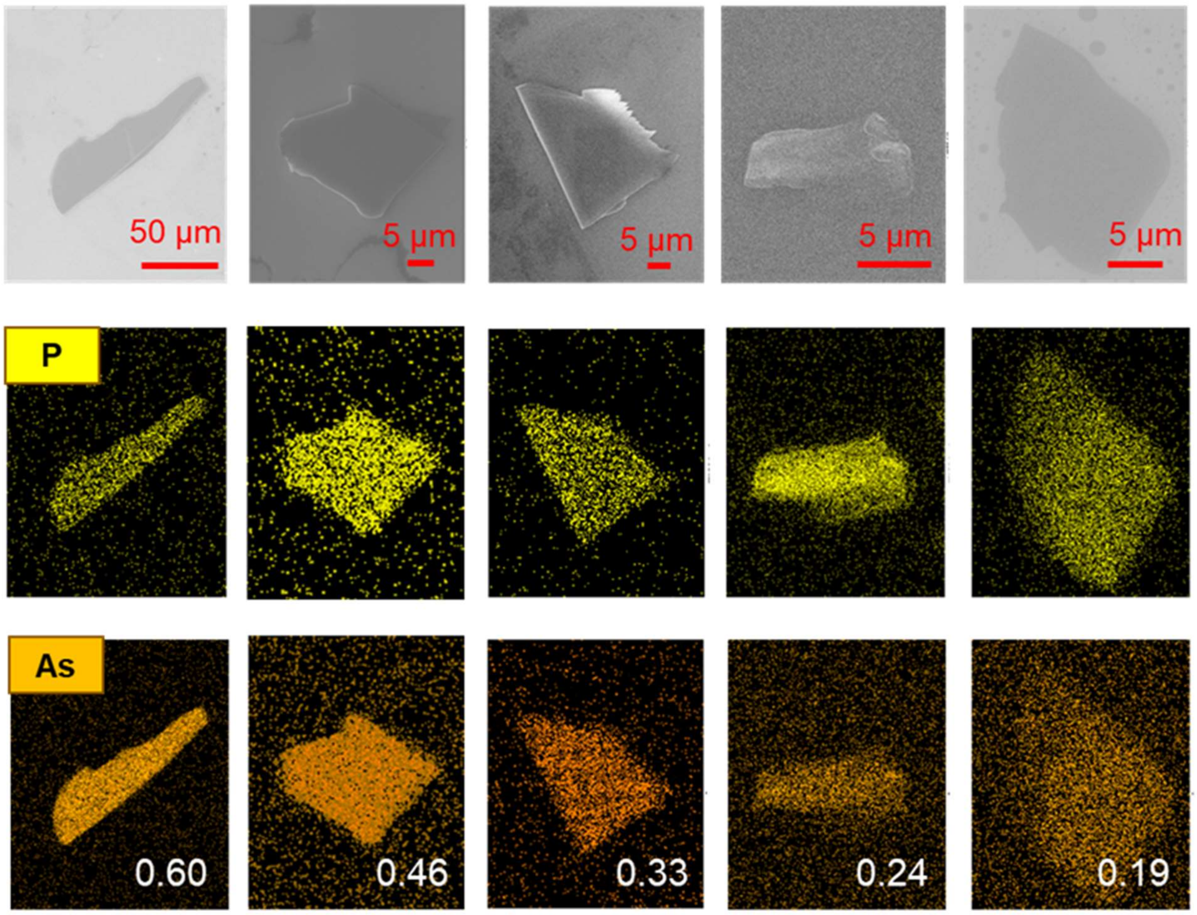

Fig. S2.

Elemental composition analysis of As_xP_{1-x} flakes with EDS measurement. The top row panels show the SEM images of example flakes on the Si/SiO_2 substrate, and the panels in the middle and bottom rows show the phosphorus (yellow) and arsenic (orange) mappings of each flake in the top panels accordingly. The exact x values of the As_xP_{1-x} samples are labeled by white numbers, in which the doping is homogeneous.

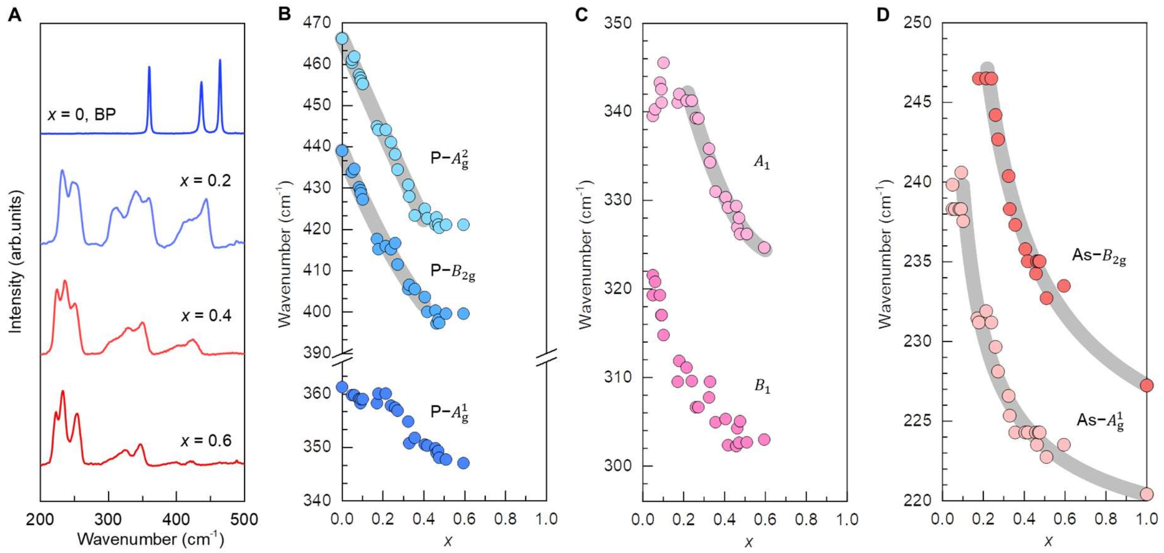

Fig. S3.

Arsenic composition dependence of Raman spectra of As_xP_{1-x}. (**A**) Raman spectra of As_xP_{1-x}, where x is approximately 0, 0.2, 0.4, and 0.6. The As concentration x was confirmed by EDS. There are three groups of Raman peaks attributed to the P-P bonds, As-P bonds and As-As bonds in As_xP_{1-x} crystals. (**B** to **D**) The x-dependent wavenumber of Raman peaks, (B) the Raman peaks contributed from the P-P bonds (blue circles), (C) the Raman peaks contributed from the As-P bonds (pink circles), and (D) the Raman peaks contributed from the As-As bonds (red circles). All these Raman peaks redshift monotonically as the x value increases. The Raman peaks of x = 1 are adapted from reference 53. The gray lines indicate the tendency of the x-dependent Raman peak, which can be used as our practical criterion for evaluating the x value.

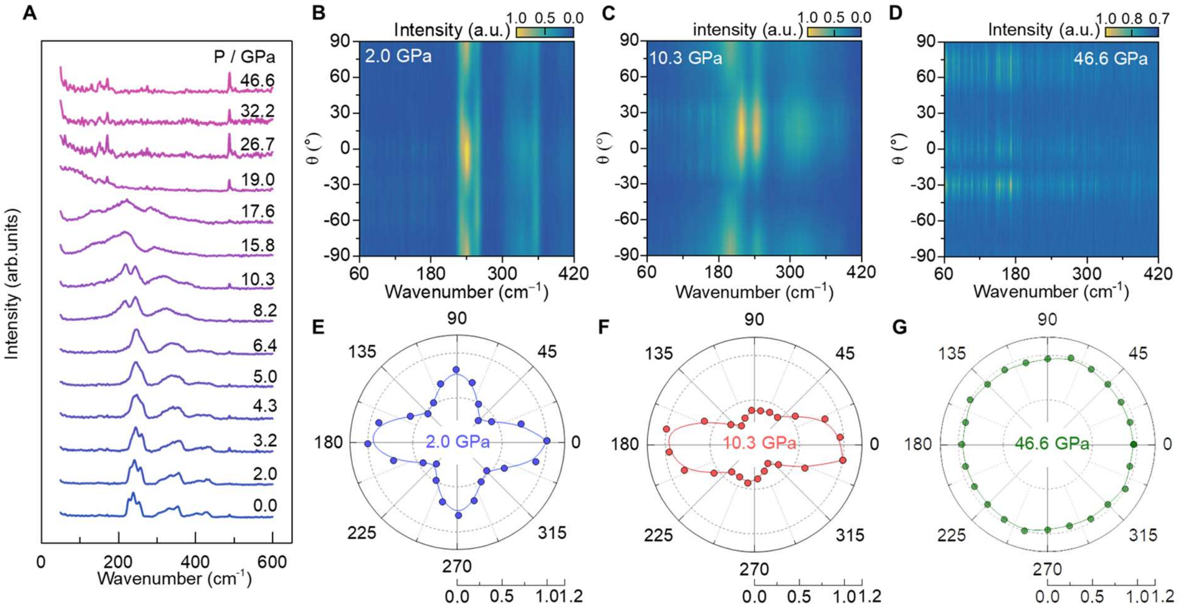

Fig. S4.

Pressure-induced evolution of Raman spectra of As_xP_{1-x}. (**A**) High-pressure Raman spectra of As_xP_{1-x} ($x = 0.36$ confirmed by Raman peak). At 8.2 GPa and 19.0 GPa, two structural transitions are recognized in the high-pressure Raman spectra of As_xP_{1-x} that correspond to the appearance of pure R and pure C structural phases. (**B** to **D**) Polarization-dependent Raman spectra of As_xP_{1-x} at (B) 2.0 GPa, (C) 10.3 GPa, and (D) 46.6 GPa. The color bar from blue to yellow indicates increased Raman intensity. (**E** to **G**) Polar plots of the intensities of Raman peaks at (E) 240 cm^{-1} for 2.0 GPa, (F) 218 cm^{-1} for 10.3 GPa, and (G) 180 cm^{-1} for 46.6 GPa.

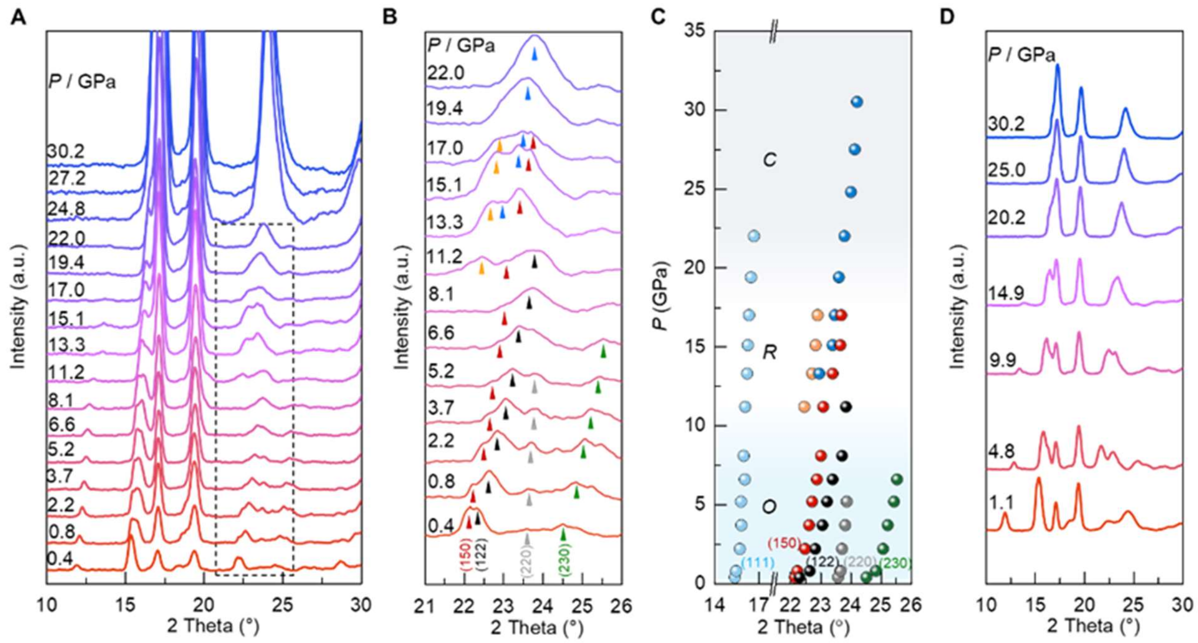

Fig. S5.

High-pressure XRD diffraction peaks of As_xP_{1-x}. (**A**) Powder XRD spectra of As_xP_{1-x} (x = 0.4) with increasing pressure. (**B**) Enlarged views of the black dotted rectangle in (A). The labeled Bragg peaks were obtained by the commercial software VESTA to simulate the powder XRD spectra of *O*-phase phosphorus and *O*-phase arsenic. The solid red, black, gray and green triangle arrows highlight these peaks of (150), (122), (220) and (230). These peaks shift and disappear with increasing pressure. Two new peaks (labeled by pink and blue triangles) appear under higher pressure. (**C**) The pressure evolution of peak positions ranges from 14° to 26°. The peak (light blue ball) is labeled orthogonal (111) by simulating the powder XRD spectrum. With increasing pressure, according to the appearance and disappearance of those peaks, one can see that the structural phase undergoes three different structures. The *O*-, *R*- and *C*-phases are shaded in light blue, white and light gray colors, respectively. (**D**) Powder XRD spectra of As_xP_{1-x} (x = 0.4) with decreasing pressure, indicating that such structural phase transitions are reversible.

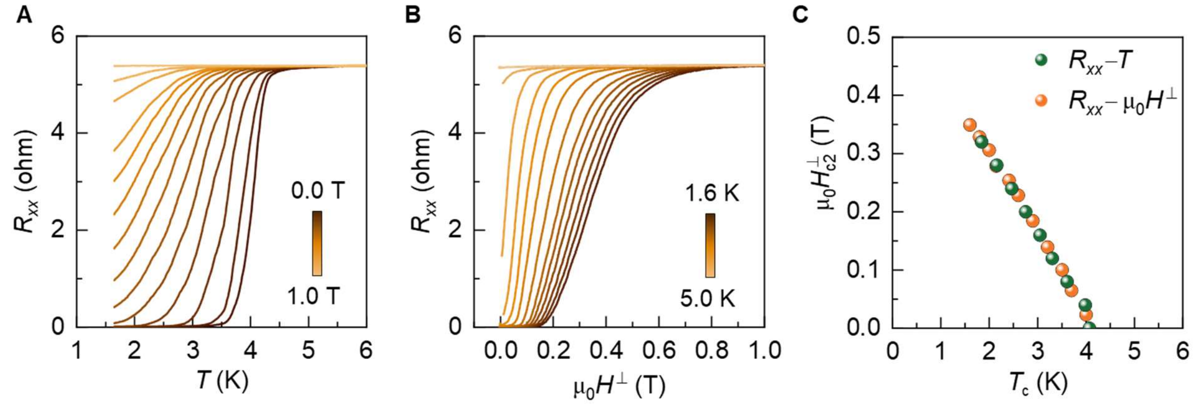

Fig. S6.

Upper critical field measurements for superconducting As_xP_{1-x}. (**A**) Magnetic field dependent $R_{xx}(T)$ curves of sample E at 25.4 GPa. (**B**) Temperature-dependent $R_{xx}(\mu_0 H^\perp)$ curves of sample E at 25.4 GPa. (**C**) The $\mu_0 H_{c2}^\perp$–T curve of superconducting sample E at 25.4 GPa, where the T_c and $H_{c2}^\perp$ values are obtained from the curves in (A) represented by green balls and (B) represented by orange balls. The $H_{c2}^\perp$ values are defined as the magnetic field at which R_{xx} becomes 50% of the normal state resistance R_N. Note that the T_c and $H_{c2}^\perp$ values obtained from two different upper critical field measurements are consistent.

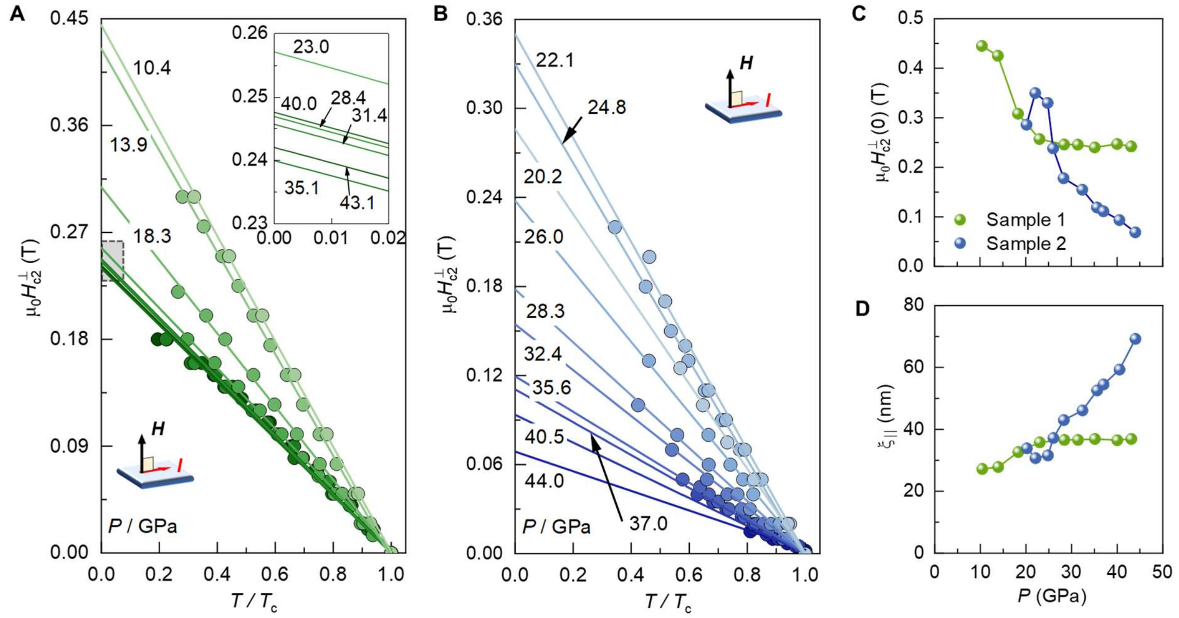

Fig. S7.

Pressure dependent $H^{\perp}_{c2}$(0) and $\xi_{\|}$(0) in As$_x$P$_{1-x}$. (**A**) Temperature-dependent $\mu_0 H^{\perp}_{c2}$ values of sample A at different pressures. The values of T are normalized by T_c and extrapolated linearly to zero temperature. The $\mu_0 H^{\perp}_{c2}(0)$ values start to be saturated with increased pressure above 18.4 GPa (gray shadow region, enlarged in the inset). (**B**) Temperature-dependent $\mu_0 H^{\perp}_{c2}$ values of sample B at different pressures. The values of T are normalized by T_c and extrapolated linearly to zero temperature. Distinct from sample A, there is no saturation behavior in $H^{\perp}_{c2}(0)$ with increased pressure in sample B with a higher x level. (**C** and **D**) Pressure-dependent $H^{\perp}_{c2}(0)$ and in-plane coherence length $\xi_{\|}(0)$ values of sample A (x = 0.23, green balls) and sample B (x = 0.65, blue balls).

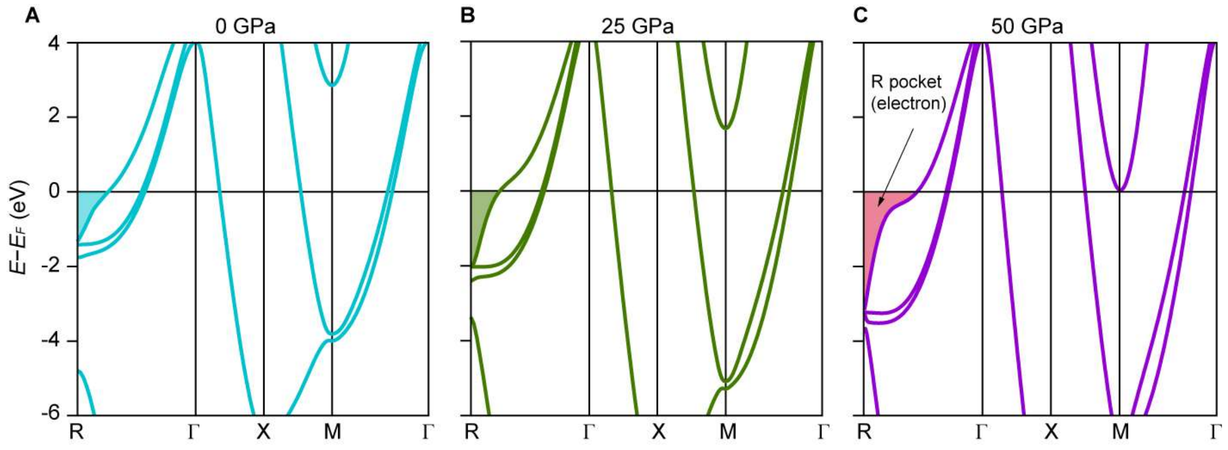

Fig. S8.

DFT calculations of As_xP_{1-x} for $x = 1$ under different pressures. The electronic band structure of cubic As at (**A**) 0 GPa (**B**) 25 GPa and (**C**) 50 GPa. The shaded area corresponds to the region occupied by the electron pocket centered at the R point in the Brillouin zone.

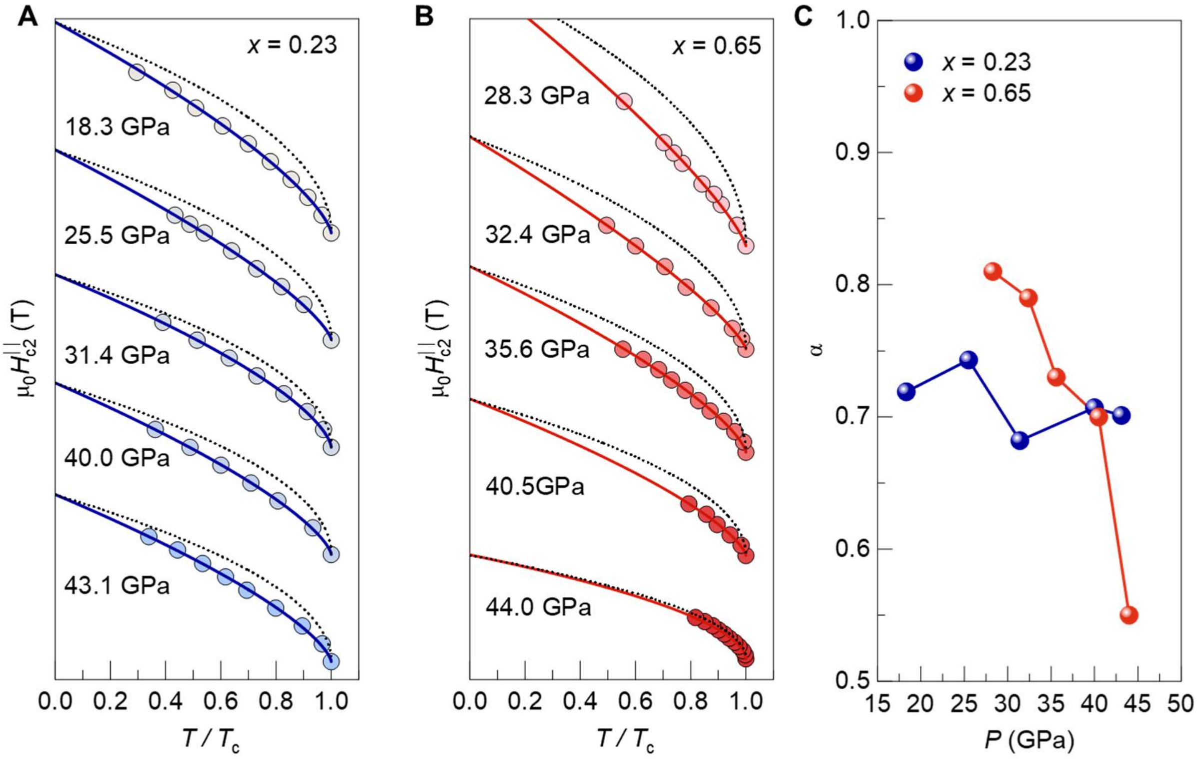

Fig. S9.

Pressure-dependent $H_{c2}^{\parallel}(T)$ with the analysis of the 2D Ginzburg–Landau model. (**A** and **B**) Temperature dependence of the in-plane upper critical field $H_{c2}^{\parallel}$ for sample A (*x*= 0.23) and sample B (*x* = 0.65). The black dashed curves are fitted to the data (blue and red circles) with $H_{c2}^{\parallel} \propto (1 - T/T_c)^{0.5}$. The blue and red solid curves are fitted with $H_{c2}^{\parallel} \propto (1 - T/T_c)^{\alpha}$. (**C**) Pressure-dependent fitting parameters α of sample A (*x* = 0.23, blue balls) and sample B (*x* = 0.65, red balls).

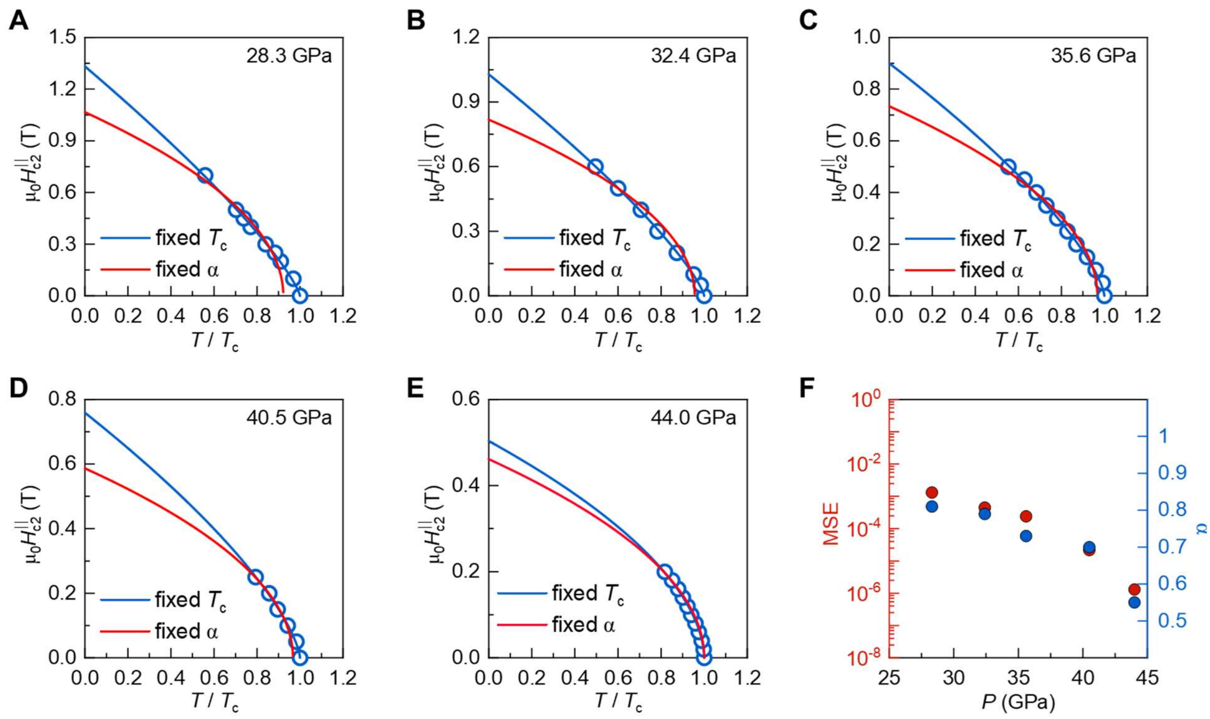

Fig. S10.

Pressure-dependent $H_{c2}^{\parallel}(T)$ with the analysis of the 2D Ginzburg–Landau model. (**A** to **E**) Temperature-dependent $\mu_0 H_{c2}^{\parallel}$ of sample B at different pressures. The red curves are the optimal fit curves based on $(1 - T/T_c)^{\alpha}$, in which the fitting parameter α is fixed at 0.5. The blue curves are the optimal fit curves based on $(1 - T/T_c)^{\alpha}$, in which the fitting parameter T_c is fixed (T_c = 1, when $H^{\parallel}$ = 0). (**F**) Pressure-dependent α fitting parameter and the mean squared error (MSE) for the optimal fit curve with fixed α = 0.5 in (A to E). The mean squared error (red circle, derived from the fitting results with red curves) decreases to the zero limit with increasing pressure, indicating the rationality of the 2D Ginzburg–Landau theory to explain 2D behavior under 44 GPa. The fitting parameter α value (blue circles, derived from the fitting results with blue curves) gradually approaches 0.5 with increasing pressure, also indicating that the dimensionality of the superconductivity gradually approaches the two-dimensional limit.

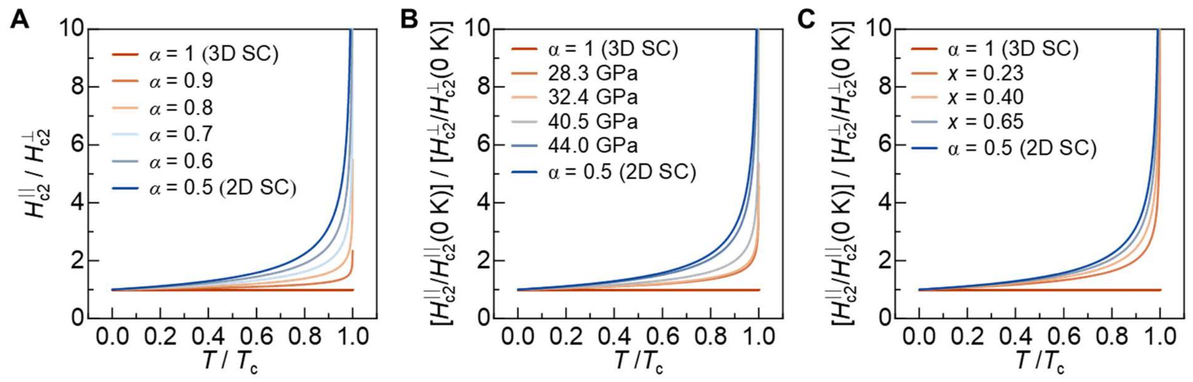

Fig. S11.

Stoichiometry- and pressure-dependent $H_{c2}^{\parallel}/H_{c2}^{\perp}$ for superconducting As_xP_{1-x}. (**A**) The fitting parameter α dependence of $H_{c2}^{\parallel}/H_{c2}^{\perp}$ based on $H_{c2}^{\parallel}/H_{c2}^{\perp} \propto (1 - T/T_c)^{\alpha-1}$, where the data are obtained from the curves in (A). (**B**) Pressure dependence of $H_{c2}^{\parallel}/H_{c2}^{\perp}$ for sample B (x = 0.65). (**C**) Stoichiometry dependence of $H_{c2}^{\parallel}/H_{c2}^{\perp}$ for sample A (x = 0.23) at 43.1 GPa, sample C (x = 0.40) at 43.0 GPa, and sample B (x = 0.65) at 44.0 GPa.

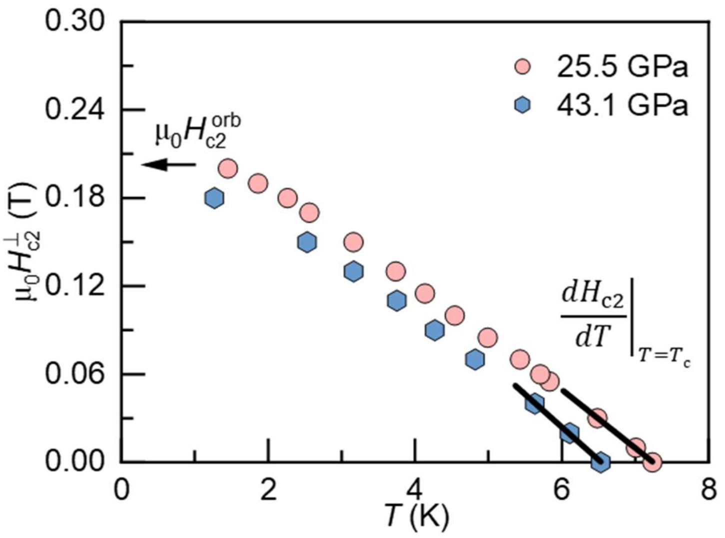

Fig. S12.

Temperature-dependent $H_{c2}^{\perp}$ with the analysis of the Werthamer-Herfand-Hohenberg model. (**A**) The pink (blue) circles represent $H_{c2}(T)$ at 25.5 GPa (43.1 GPa) for sample A (x = 0.23). The upper critical field H_{c2} is defined at 50% of the normal-state resistance drop. The black curves represent a fitted line of $H_{c2}(T)$ close to T_c. The black arrow corresponds to the orbital limited field $\mu_0 H_{c2}^{orb}(0) = 0.693 T_c \left.\frac{dH_{c2}}{dT}\right|_{T=T_c} \approx 0.2$ T, where the 0.693 is for the dirty limit.

Table S1. Parameters of superconducting phase and WHH fits for Sample A

Pressure (GPa)	T_c (K)	$\left.\frac{dH_{c2}}{dT}\right|_{T=T_c}$ (T/K)	$\mu_0 H_{c2}^{orb}$ (T)	$\mu_0 H_{c2}^{Pauli}$ (T)	Maki parameter α_M
18.3	6.73	0.045	0.212	12.383	0.024
25.5	7.24	0.040	0.199	13.322	0.021
31.4	7.14	0.040	0.201	13.138	0.022
40.0	6.74	0.042	0.197	12.402	0.023
43.1	6.53	0.045	0.202	12.015	0.024

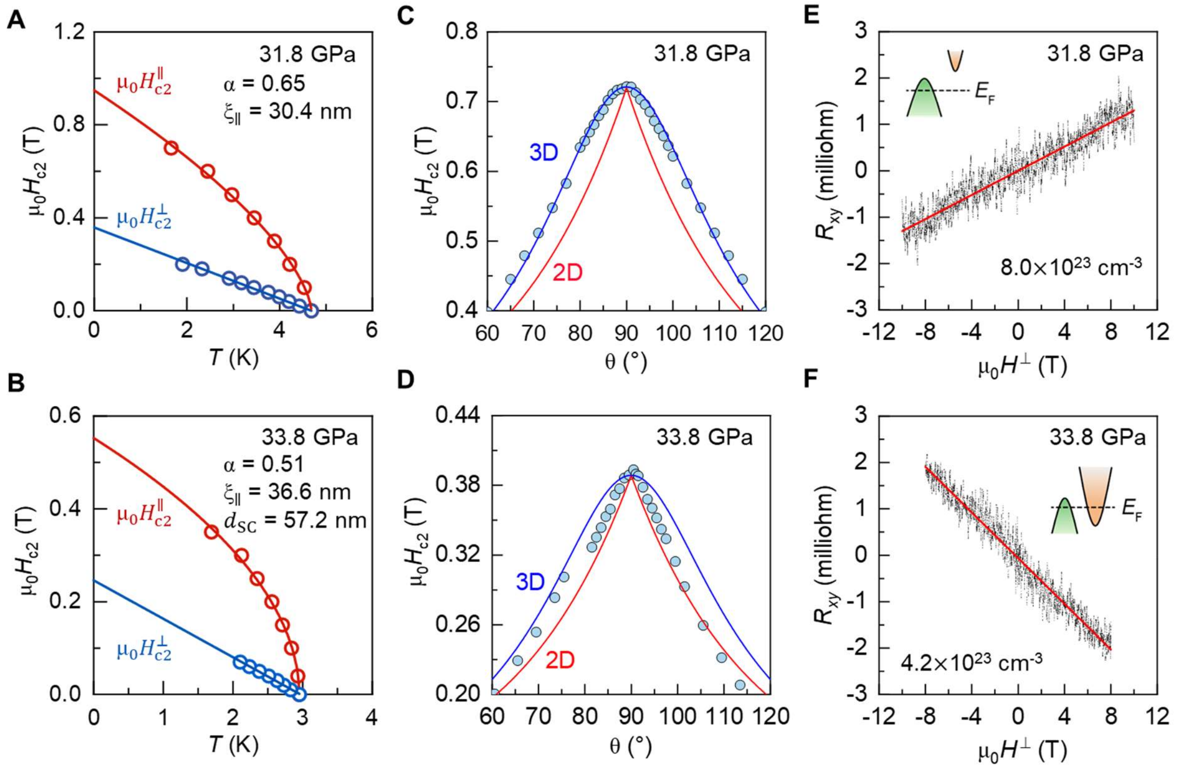

Fig. S13.

Pressure-induced evolution of the superconductivity dimensionality and carrier density in cubic As$_x$P$_{1-x}$. (**A** and **B**) Temperature dependence of both $H_{c2}^{\parallel}$ (labeled by hollow red circles) and $H_{c2}^{\perp}$ (labeled by hollow blue circles) for sample D at 31.8 GPa and 33.8 GPa. The fitted parameters of the α, $\xi_{\parallel}$ and d_{SC} values are marked inside each panel. (**C** and **D**) Angle dependence of H_{c2} values at 1.5 K and fitting curves based on the 3D Ginzburg–Landau model (blue curves) and 2D Tinkham model (red curves). The fitting parameters $H_{c2}^{\perp}$ and $H_{c2}^{\parallel}$ are obtained by $H_{c2}(T)$ in (A) and (B). The cusp-shaped peak becomes more pronounced with increasing pressure, indicating the evolution from 3D to 2D superconductivity. (**E** and **F**) Pressure-dependent Hall resistance R_{xy} and the resulting carrier density n_{3D} at 10 K. Insets show the evolution of the valleys in the band structure crossing the Fermi level with increasing pressure. The R_{xy} values are obtained by antisymmetrizing the raw data, and the n_{3D} values are obtained by linear fitting of the $R_{xy}(\mu_0 H^{\perp})$ data. The result shows a transition from hole-to-electron conducting behavior at 33.8 GPa.

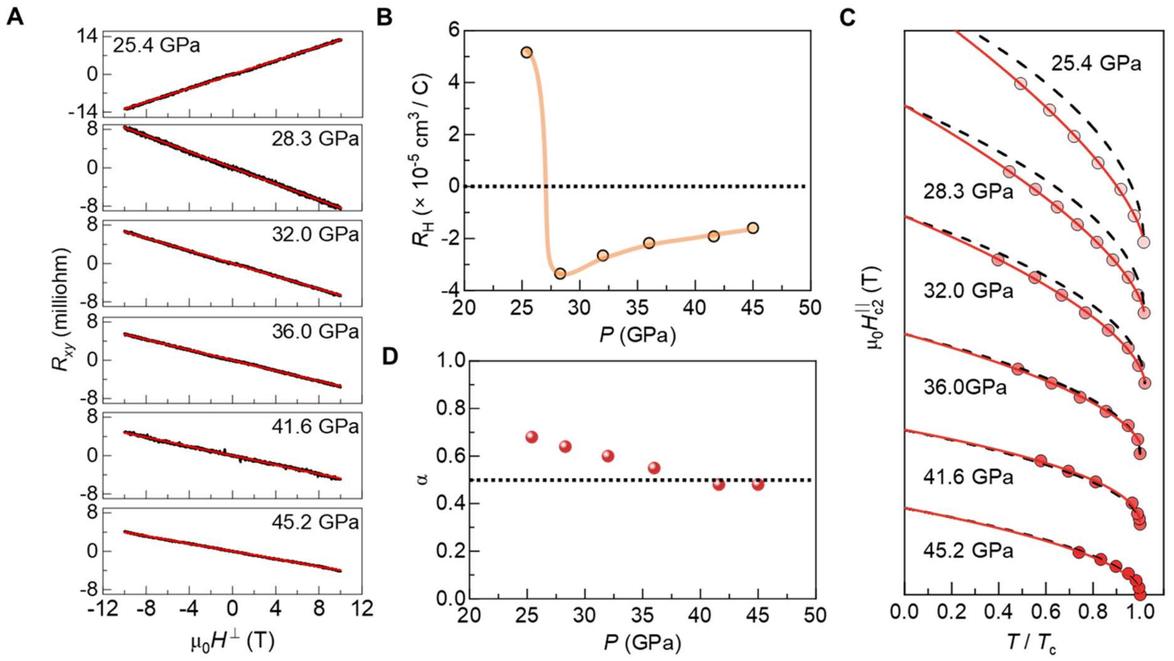

Fig. S14.

$H^{\parallel}_{c2}(T)$ with the analysis of the 2D Ginzburg–Landau model and the value of R_H under different pressures. (**A**) Pressure-dependent Hall resistance R_{xy} for sample E (x = 0.58, 41-nm-thick) at 10 K. The R_{xy} values are obtained by antisymmetrizing the raw data. The red fitting curves are based on $R_{xy} = \frac{R_H}{d}\mu_0 H = \frac{1}{ned}\mu_0 H$, where R_H, n, e, d and $\mu_0 H$ are the Hall coefficient, carrier density, the elementary charge (1.6×10^{-19} C), sample thickness and magnetic field, respectively. (**B**) The pressure-dependent Hall coefficient R_H for sample E (x = 0.58, 41-nm-thick). The circles represent the experimental data, and the solid line indicates the tendency of the pressure-dependent R_H. (**C**) Temperature dependence of the in-plane upper critical field $H^{\parallel}_{c2}$ of sample E (x = 0.58, 41-nm-thick). The red curves are the optimal fit curves based on $H^{\parallel}_{c2} \propto (1 - T/T_c)^{\alpha}$, and the black dashed lines are the fit curves based on $H^{\parallel}_{c2} \propto (1 - T/T_c)^{0.5}$. (**D**) Pressure-dependent fitting parameters α in (C). The black dashed line represents α = 0.5, implying 2D superconducting behavior.

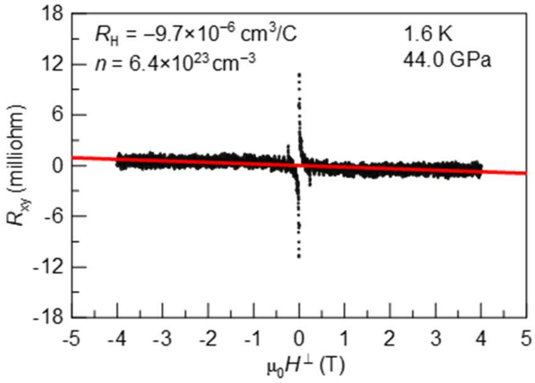

Fig. S15.

The Hall data for sample B (*x* = 0.65, 53-nm-thick) at 44.0 GPa. The R_{xy} values are obtained by anti-symmetrizing the raw data. The red fitting curve is based on $R_{xy} = \frac{R_\mathrm{H}}{d} \mu_0 H = \frac{1}{ned} \mu_0 H$, where R_H, n, e, d and $\mu_0 H$ are the Hall coefficient, carrier density, elementary charge (1.6×10^{-19} C), sample thickness and magnetic field, respectively.

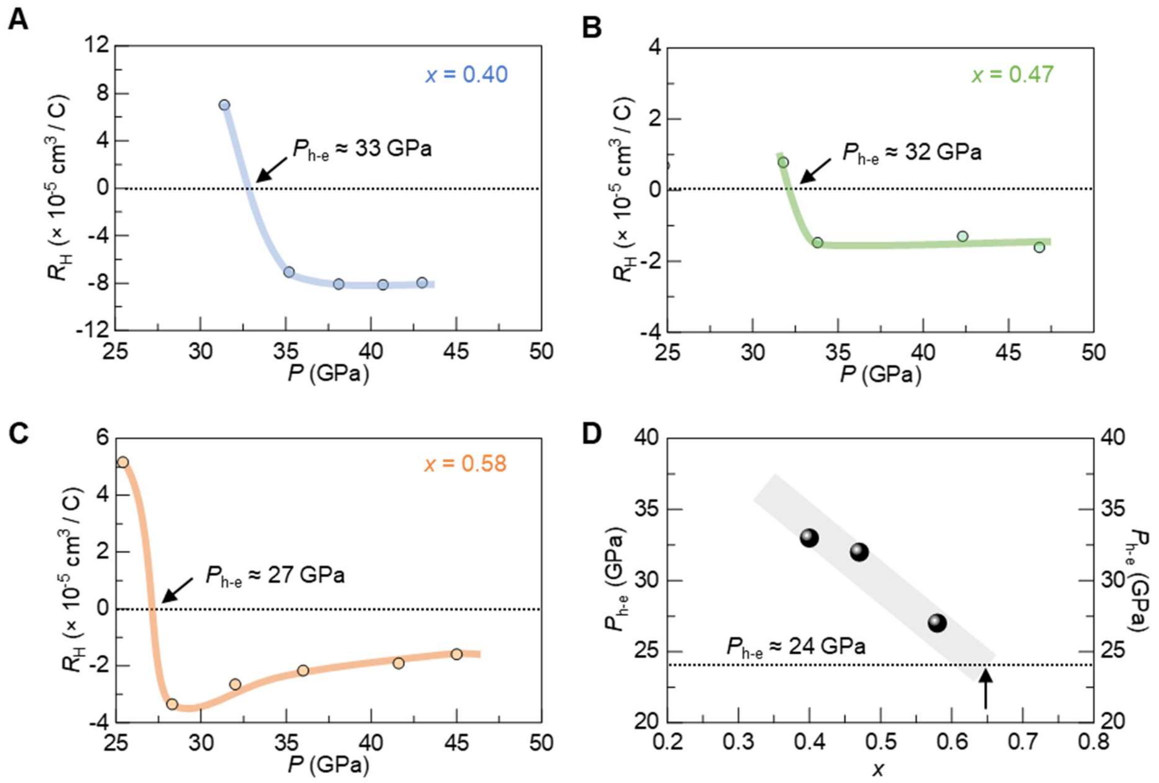

Fig. S16.

Analysis of the Hall effect in As_xP_{1-x}. (**A to C**) The pressure-dependent Hall coefficient R_H for sample C (x = 0.40), sample D (x = 0.47) and sample E (x = 0.58). The circles represent the experimental data, and the solid line indicates the tendency of the pressure-dependent R_H, which can be used to estimate P_{h-e} (the critical pressure when the sign R_H changes from position to negative). (**D**) The stoichiometry-dependent P_{h-e}. The black balls represent the estimated value of P_{h-e} from (**A**) to (**C**). The solid line indicates the tendency of the stoichiometry-dependent P_{h-e}. The value of P_{h-e} for sample B (x = 0.65) is estimated at 24 GPa.

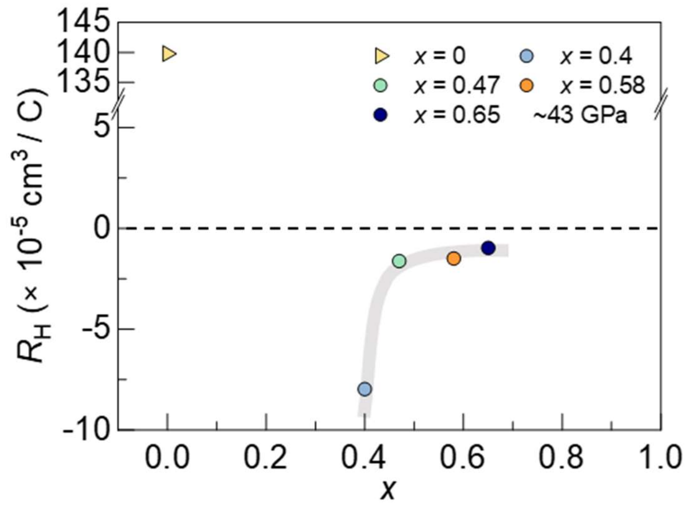

Fig. S17.

The stoichiometry-dependent Hall coefficient R_H at about 43 GPa. The black dashed line indicates that the boundary of the major carrier type varies from hole to electron. The data of yellow triangle represents R_H for $x = 0$ adopted from reference 34. The data for x=0.4 and x = 0.65 were obtained at 1.6 K, and the data for x = 0.47 and x = 0.58 were obtained at 10 K.

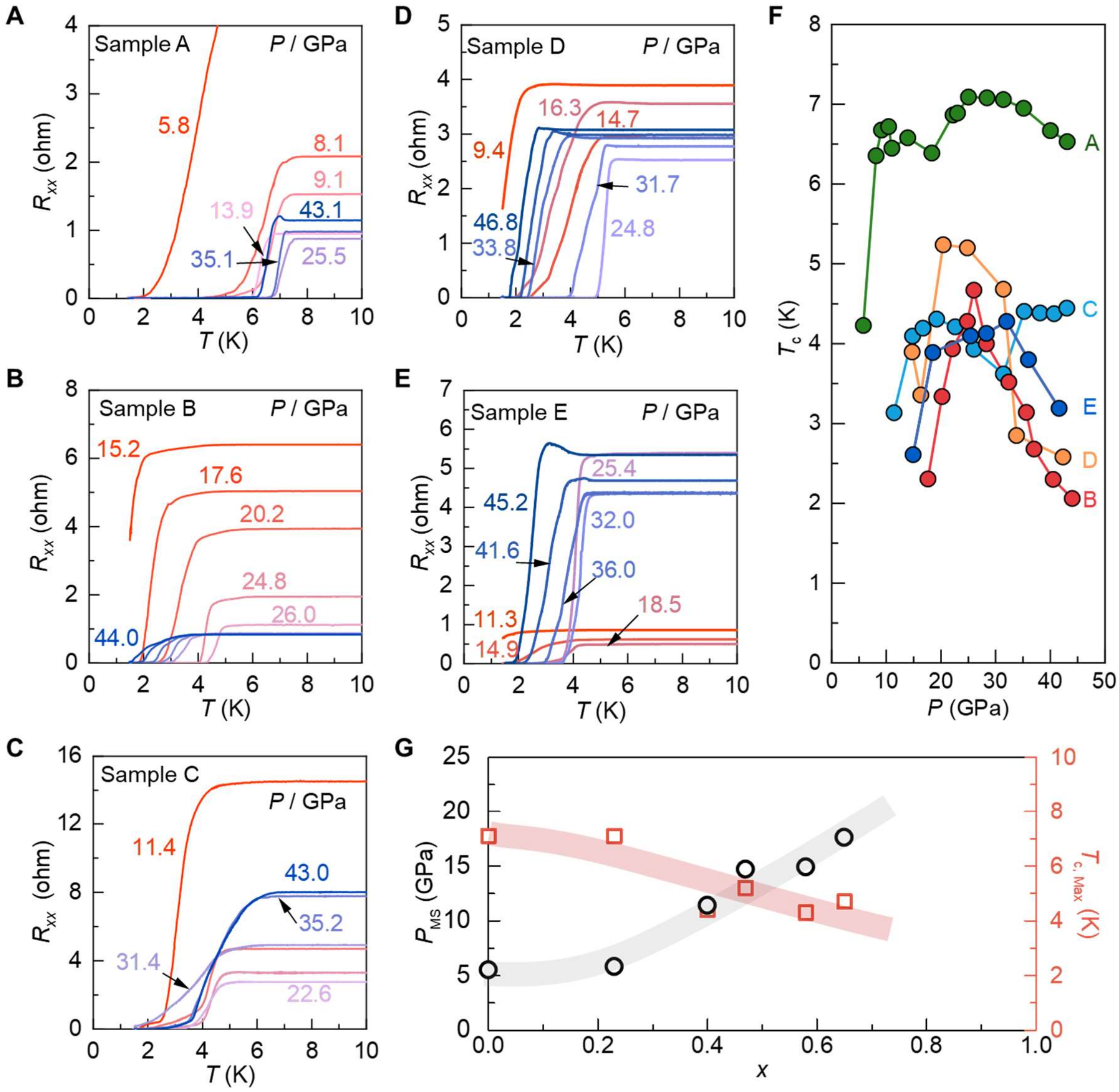

Fig. S18.

Stoichiometry-dependent superconducting phase diagram of As_xP_{1-x}. (**A** to **E**) The pressure-dependent $R_{xx}(T)$ curves for samples A (x = 0.23), B (x = 0.65), C (x = 0.40), D (x = 0.47) and E (x = 0.58). (**F**) Pressure-dependent T_c values for the above samples. T_c is defined as the temperature at which R_{xx} becomes 50% of the normal state resistance R_N. Dome-shaped superconducting behavior is achieved in all samples (distinguished by color). (**G**) Stoichiometry-dependent P_{MS} (black circles) and $T_{c,Max}$ (red squares). With increasing As concentration, P_{MS} moves toward a higher value, while the corresponding $T_{c,Max}$ decreases. The data points at x = 0 are adopted from reference 31.

Table S2. Superconducting critical temperature of As_xP_{1-x} samples.

Sample No.	x	P_{MS} (GPa)	T_c at P_{MS} (K)	P with $T_{c,Max}$ (GPa)	$T_{c,Max}$ (K)
A	0.23	5.8	4.2	25.5	7.1
B	0.65	17.6	2.3	26.0	4.7
C	0.40	11.4	3.1	35.2	4.4
D	0.47	14.7	3.9	24.8	5.2
E	0.58	14.9	2.6	32.0	4.3

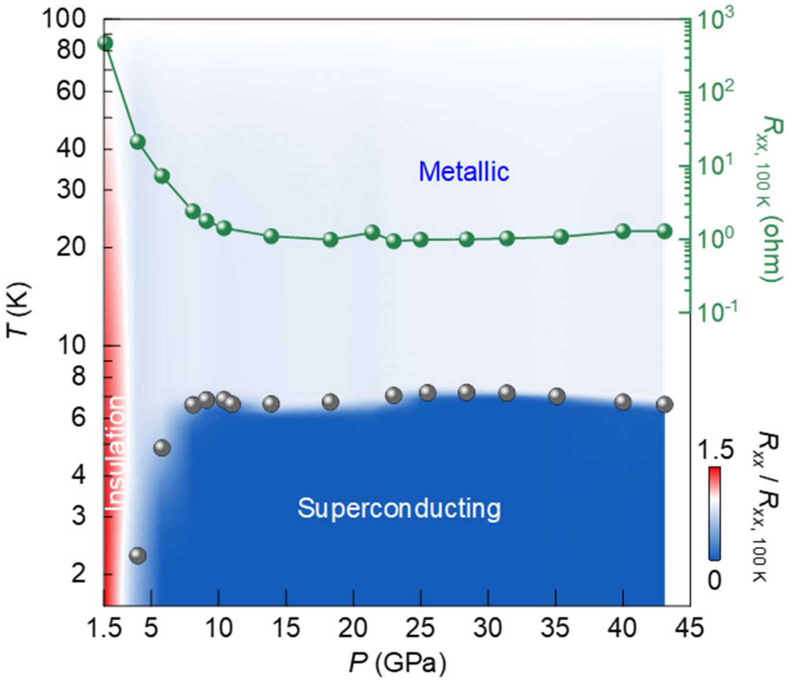

Fig. S19.

The superconducting phase diagram of sample A with colored mapping of normalized resistance $R_{xx}/R_{xx,100\,K}$. Three distinct regions are shown: the insulating region (shaded in red), metallic region (shaded in light blue), and superconducting region (shaded in blue). The gray spheres represent the superconducting critical temperature T_c, which is defined as the temperature where the resistance drops to 50% of its normal-state resistance at 10 K. The pressure-dependent $R_{xx,100\,K}$ is shown by green spheres.

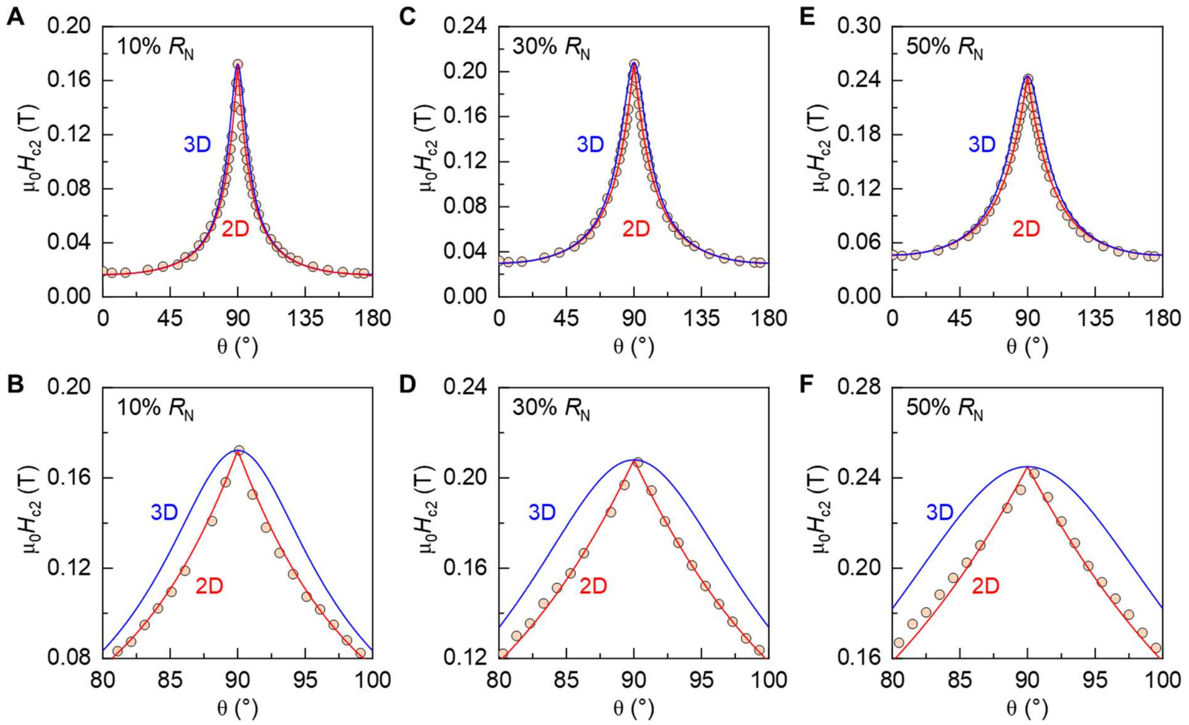

Fig. S20.

The robustness of the 2D nature of superconductivity and the solid validity of the analysis method. (**A** to **F**) Angular dependence of the upper critical fields $H_{c2}(\theta)$ of sample D at 46.8 GPa with different definitions of upper critical fields at 1.5 K. The data for $H_{c2}(\theta)$ can be fitted with the 2D Tinkham mode (red line) rather than the 3D Ginzburg–Landau model (blue line). H_{c2} is defined as the magnetic field, where R_{xx} becomes 10%R_{N} (A and B), 30%R_{N} (C and D) and 50%R_{N} (E and F). Panels (B), (D) and (F) are enlargements of (A), (C) and (E). The 2D superconductivity is robust to different definitions of upper critical fields.

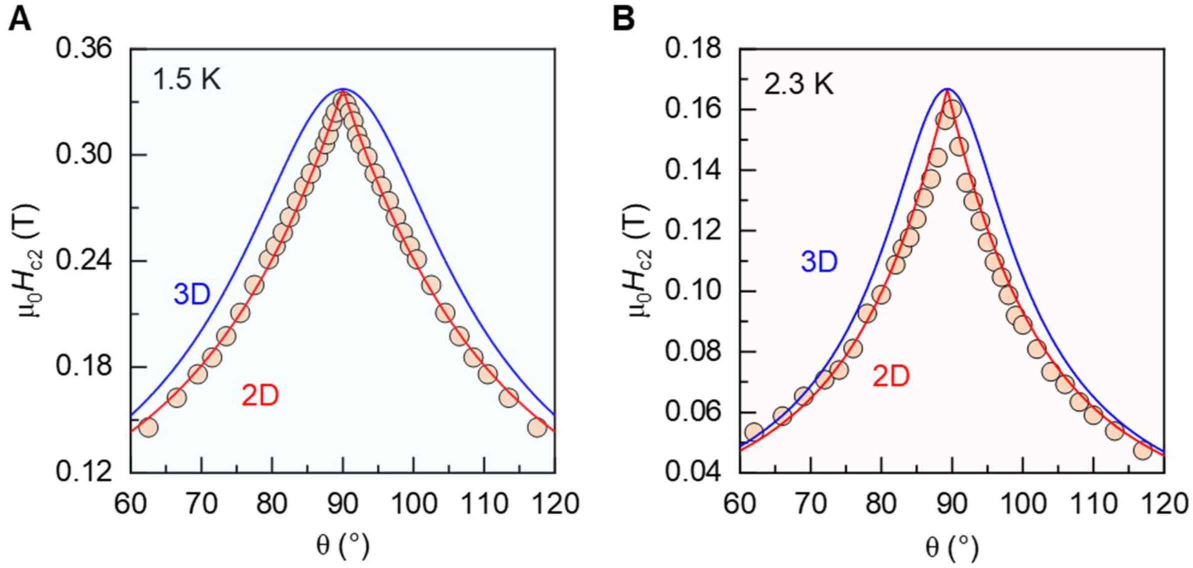

Fig. S21.

The robustness of the 2D nature of superconductivity at different temperatures below T_c. (**A** and **B**) Angular dependence of $H_{c2}(\theta)$ of sample D (42.3 GPa) at different temperatures: (A) 1.5 K and (B) 2.3 K. The data for $H_{c2}(\theta)$ can be fitted with the 2D Tinkham model (red curve) at θ = 90 ± 30° rather than the 3D Ginzburg–Landau model (blue line).

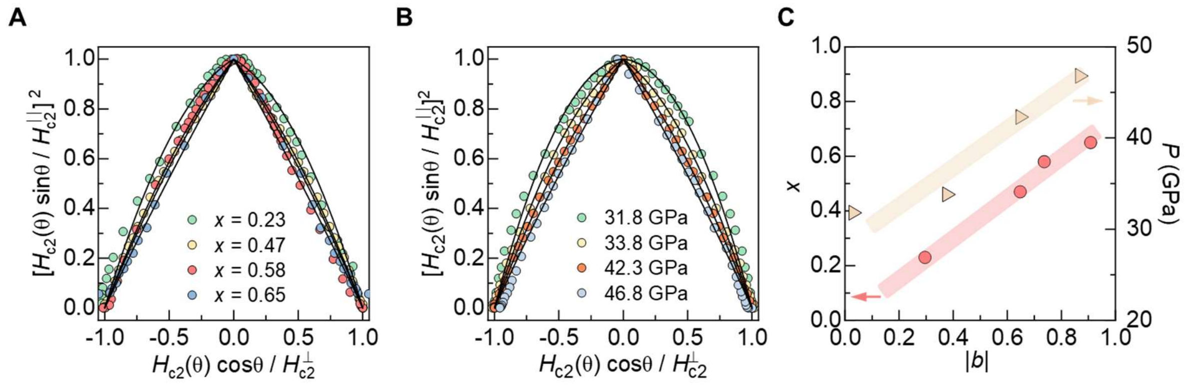

Fig. S22.

Stoichiometry- and pressure-dependent superconducting dimensionality in As_xP_{1-x}. (**A**) $H_{c2}(\theta)$ curves at different stoichiometries x plotted in an appropriate dimensionless form near 43 GPa. (**B**) $H_{c2}(\theta)$ curves at different pressures plotted by an appropriate dimensionless form for sample D (x = 0.47). The circles represent the experimental data points. The black solid line is the fitting curve by $\left[\frac{H_{c2}(\theta)\sin\theta}{H_{c2}^{\parallel}}\right]^2 = a\left[\frac{H_{c2}(\theta)\cos\theta}{H_{c2}^{\perp}}\right]^2 + b\left|\frac{H_{c2}(\theta)\cos\theta}{H_{c2}^{\perp}}\right| + 1$. (**C**) The stoichiometry- and pressure-dependent value of exponent $|b|$. The value of $|b|$ represents the relative contributions of the 2D Tinkham model. The lines indicate the tendency of the $|b|$-dependent stoichiometry x and pressure, which can be used as our practical criterion for evaluating superconducting dimensionality.

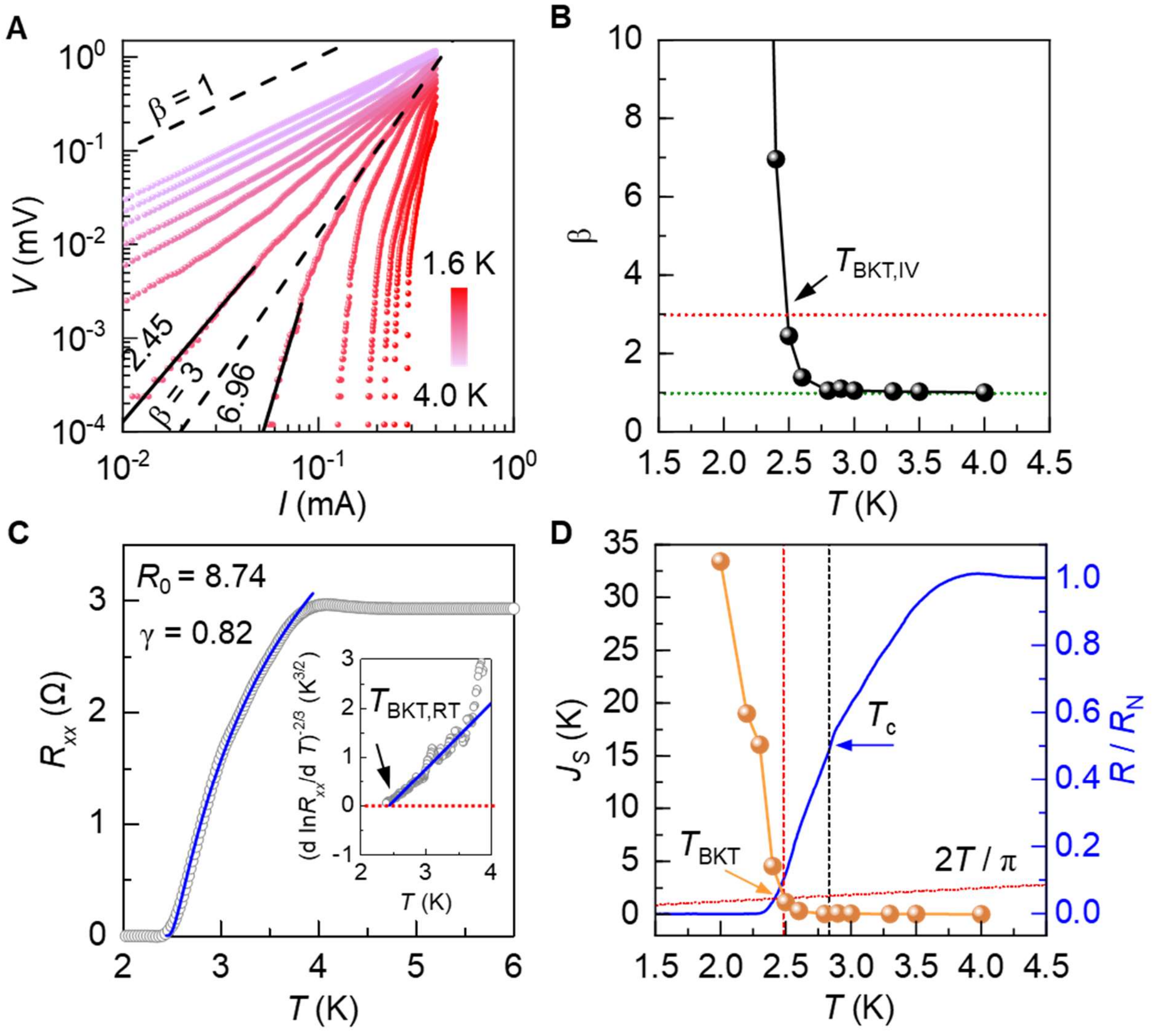

Fig. S23.

BKT analysis for 2D superconductivity in As_xP_{1-x}. (**A**) Logarithmic plots of the $V(I)$ curves at different temperatures for sample D at 33.8 GPa. The black solid line is the fitting curve by $V \propto I^{\beta}$, and the black dashed line indicates the relation of $V \propto I^3$. (**B**) The temperature-dependent value of exponent β near T_{BKT} at 33.8 GPa. $T_{BKT,IV}$ = 2.48 K at 33.8 GPa. (**C**) Temperature dependence of the resistance at 33.8 GPa. The value of $T_{BKT,RT}$ obtained by equation S1 is 2.46 K at 33.8 GP. The blue curve is the best-fit line, and the fitting parameters are marked in the figure. The inset shows the linear temperature dependence around the BKT transition temperature by constructing the scaling plot with $(\frac{\mathrm{d}\ln\ \ _{xx}}{\mathrm{d}T})^{-\frac{2}{3}}$. (**D**) The temperature-dependent value of superfluid stiffness J_S at 33.8 GPa by $J_S[\mathrm{K}] = (\beta - 1)\frac{T}{\pi}$ compared with the normalized resistivity R/R_N (right axis). T_{BKT} is the temperature corresponding to the intersection point of the universal $2T/\pi$ BKT curve and $J_S - T$ curve (red dashed lines). The superfluid stiffness reaches 0 above T_c, and T_c is defined as the temperature where the resistance drops to 50% of its normal-state resistance at 10 K (black dashed line).

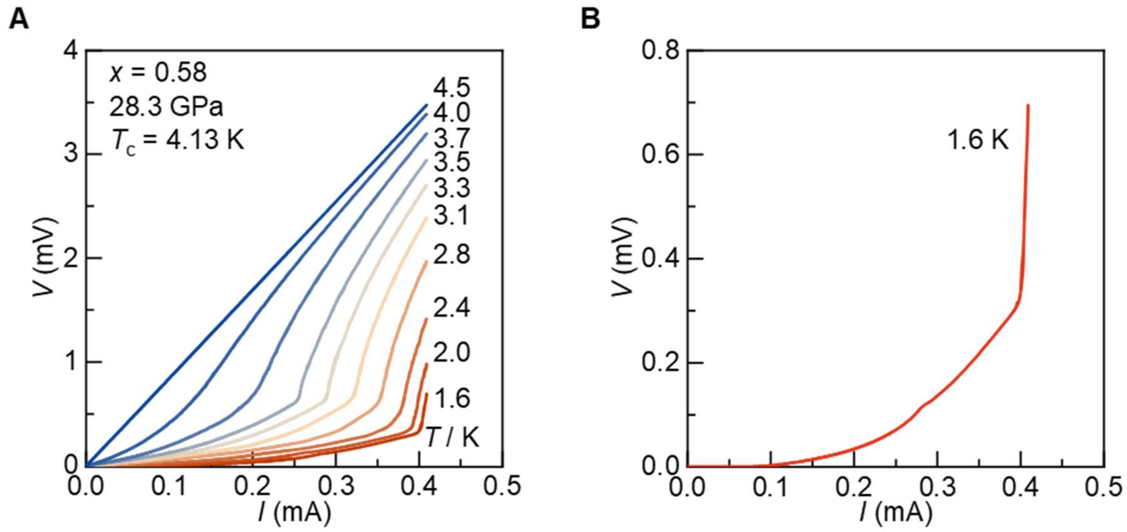

Fig. S24.

Temperature-dependent $I - V$ measurements in As_xP_{1-x}. (**A**) $I - V$ curves measured at different temperatures for sample E at 28.3 GPa. (**B**) An abrupt jump in the $I - V$ curve near the critical current of ~ 0.4 mA at 1.6 K.

Table S3. Survey of the superconducting materials in Fig. 4H.

Compound	d (nm)	ξ_{GL} (nm)	Dimensionality (2D or 3D)	Reference
Sample A	56	36.9	3D	This work
Sample B	53	69.2	2D	This work
Sample C	59	39.1	2D+3D	This work
Sample D	63	49.1	2D	This work
2H-TaS_2	2.22	20	2D	(*64*)
2H-$NbSe_2$	3.97	10	2D	(*64*)
2H-$NbSe_2$	Bulk	7.4	3D	(*65*)
$MoTe_2$	9.13	19.7	2D	(*66*)
WTe_2	~0.44	80	2D	(*19*)
$PdTe_2$	~2.0	26	2D	(*67*)
MoS_2	1.5	8	2D	(*5*)
1T-MoS_2	12.1	11.9	2D	(*68*)
TaS_2	4.2	13.9	2D	(*15*)
TaS_2	5.8	19.1	2D	(*15*)
TaS_2	Bulk	54.7	3D	(*15*)
α-MoGe film	7	34.2	2D	(*69*)
Pb film	0.3	49	2D	(*21*)
Pb film	~2	74	2D	(*70*)
In film	~3	25	2D	(*70*)
NbN film	4	8.6	2D	(*71*)
NbN film	50	2	3D	(*71*)
NbN film	100	2.14	3D	(*71*)
Nb film	2	10.4	2D	(*41*)
Nb film	17	7.7	2D	(*41*)
Nb film	33	11.3	3D	(*72*)
Nb film	92	11.5	3D	(*72*)
α-InO_x	30	8.37	3D	(*73*)
TBG	0.6	52	2D	(*17*)
$LaAlO_3/SrTiO_3$	~3.2	70	2D	(*22*)
$LaAlO_3/SrTiO_3$	~6	105	2D	(*22*)
$LaAlO_3/SrTiO_3$	24	44	2D	(*24*)
$LaAlO_3/KTaO_3$	7.7	41	2D	(*74*)
$LaAlO_3/KTaO_3$	8	27.3	2D	(*74*)
$EuO/KTaO_3$	5.1	13	2D	(*75*)
ZrNCl	1.8	12.8	2D	(*30*)
ZrN	7.8	12.1	2D	(*76*)
Bi_2Te_3/FeTe	7	5.2	2D	(*77*)
$FeSe/SrTiO_3$	0.55	2.45	2D	(*78, 79*)
FeSe/MgO	0.6	2	2D	(*80*)
$SbTe_2/Fe_{1+y}Te$	3.9	4.1	2D	(*81*)
FeSe	bulk	4.54	3D	(*82*)
FeS	bulk	34.3	3D	(*83*)
BaFeCoAs	bulk	2.61	3D	(*84*)

Compound	d (nm)	ξ_{GL} (nm)	Dimensionality (2D or 3D)	Reference
BaFeAsP	bulk	32	3D	(*85*)
$KFeSe_2$	bulk	2.3	3D	(*86*)
LiFeAs	bulk	4.8	3D	(*87*)
FeTeSe	bulk	2.68	3D	(*88*)
Sr_2RuO_4	bulk	91	3D	(*89*)
V_3Si	bulk	4.19	3D	(*90*)
Bi_4I_4	bulk	11.5	3D	(*91*)
$SrAs_3$	bulk	12.9	3D	(*92*)
CrAs	bulk	18.5	3D	(*93*)
Li	bulk	10.5	3D	(*94*)
UGe_2	bulk	12.8	3D	(*95*)
CsV_3Sb_5	bulk	19.0	3D	(*96*)
BP	bulk	68.5	3D	(*31*)

Note: TBG is for twisted bilayer graphene, BaFeAsP is for $BaFe_2(As_{0.68}P_{0.32})_2$, FeTeSe is for $Fe_{1+y}Te_{1-x}Se_x$, BP is for black phosphorus.

REFERENCES AND NOTES

1. J. Wu, A. T. Bollinger, X. He, I. Božović, Spontaneous breaking of rotational symmetry in copper oxide superconductors. *Nature* **547**, 432–435 (2017).

2. L. Nie, K. Sun, W. Ma, D. Song, L. Zheng, Z. Liang, P. Wu, F. Yu, J. Li, M. Shan, D. Zhao, S. Li, B. Kang, Z. Wu, Y. Zhou, K. Liu, Z. Xiang, J. Ying, Z. Wang, T. Wu, X. Chen, Charge-density-wave-driven electronic nematicity in a kagome superconductor. *Nature* **604**, 59–64 (2022).

3. S. Kasahara, H. J. Shi, K. Hashimoto, S. Tonegawa, Y. Mizukami, T. Shibauchi, K. Sugimoto, T. Fukuda, T. Terashima, A. H. Nevidomskyy, Y. Matsuda, Electronic nematicity above the structural and superconducting transition in $BaFe_2(As_{1-x}P_x)_2$. *Nature* **486**, 382–385 (2012).

4. J. Falson, Y. Xu, M. Liao, Y. Zang, K. Zhu, C. Wang, Z. Zhang, H. Liu, W. Duan, K. He, H. Liu, J. H. Smet, D. Zhang, Q. K. Xue, Type-II Ising pairing in few-layer stanene. *Science* **367**, 1454–1457 (2020).

5. Y. Saito, Y. Nakamura, M. S. Bahramy, Y. Kohama, J. Ye, Y. Kasahara, Y. Nakagawa, M. Onga, M. Tokunaga, T. Nojima, Y. Yanase, Y. Iwasa, Superconductivity protected by spin–Valley locking in ion-gated MoS_2. *Nat. Phys.* **12**, 144–149 (2015).

6. X. Xi, Z. Wang, W. Zhao, J.-H. Park, K. T. Law, H. Berger, L. Forró, J. Shan, K. F. Mak, Ising pairing in superconducting $NbSe_2$ atomic layers. *Nat. Phys.* **12**, 139–143 (2015).

7. Y. Liu, Y. Xu, J. Sun, C. Liu, Y. Liu, C. Wang, Z. Zhang, K. Gu, Y. Tang, C. Ding, H. Liu, H. Yao, X. Lin, L. Wang, Q. K. Xue, J. Wang, Type-II ising superconductivity and anomalous metallic state in macro-size ambient-stable ultrathin crystalline films. *Nano Lett.* **20**, 5728–5734 (2020).

8. L. Jiao, S. Howard, S. Ran, Z. Wang, J. O. Rodriguez, M. Sigrist, Z. Wang, N. P. Butch, V. Madhavan, Chiral superconductivity in heavy-fermion metal UTe_2. *Nature* **579**, 523–527 (2020).

9. S. Ran, C. Eckberg, Q. P. Ding, Y. Furukawa, T. Metz, S. R. Saha, I. L. Liu, M. Zic, H. Kim, J. Paglione, N. P. Butch, Nearly ferromagnetic spin-triplet superconductivity. *Science* **365**, 684–687 (2019).

10. I. M. Hayes, D. S. Wei, T. Metz, J. Zhang, Y. S. Eo, S. Ran, S. R. Saha, J. Collini, N. P. Butch, D. F. Agterberg, A. Kapitulnik, J. Paglione, Multicomponent superconducting order parameter in UTe_2. *Science* **373**, 797–801 (2021).

11. L. Kong, S. Zhu, M. Papaj, H. Chen, L. Cao, H. Isobe, Y. Xing, W. Liu, D. Wang, P. Fan, Y. Sun, S. Du, J. Schneeloch, R. Zhong, G. Gu, L. Fu, H.-J. Gao, H. Ding, Half-integer level shift of vortex bound states in an iron-based superconductor. *Nat. Phys.* **15**, 1181–1187 (2019).

12. M. Tinkham, *Introduction to Superconductivity* (Courier Corporation, 2004).

13. V. J. Emery, S. A. Kivelson, Importance of phase fluctuations in superconductors with small superfluid density. *Nature* **374**, 434–437 (1995).

14. Y. Guo, Y. F. Zhang, X. Y. Bao, T. Z. Han, Z. Tang, L. X. Zhang, W. G. Zhu, E. G. Wang, Q. Niu, Z. Q. Qiu, J. F. Jia, Z. X. Zhao, Q. K. Xue, Superconductivity modulated by quantum size effects. *Science* **306**, 1915–1917 (2004).

15. E. Navarro-Moratalla, J. O. Island, S. Manas-Valero, E. Pinilla-Cienfuegos, A. Castellanos-Gomez, J. Quereda, G. Rubio-Bollinger, L. Chirolli, J. A. Silva-Guillen, N. Agrait, G. A. Steele, F. Guinea, H. S. van der Zant, E. Coronado, Enhanced superconductivity in atomically thin TaS_2. *Nat. Commun.* **7**, 11043 (2016).

16.. S. L. Chun, G.-G. Zheng, J. L. Vincent, I. K. Schuller, Dimensional crossover in superlattice superconductors. *Phys. Rev. B* **29**, 4915–4920 (1984).

17. Y. Cao, V. Fatemi, S. Fang, K. Watanabe, T. Taniguchi, E. Kaxiras, P. Jarillo-Herrero, Unconventional superconductivity in magic-angle graphene superlattices. *Nature* **556**, 43–50 (2018).

18. L. J. Li, E. C. O'Farrell, K. P. Loh, G. Eda, B. Ozyilmaz, A. H. Castro Neto, Controlling many-body states by the electric-field effect in a two-dimensional material. *Nature* **529**, 185–189 (2016).

19. V. Fatemi, S. Wu, Y. Cao, L. Bretheau, Q. D. Gibson, K. Watanabe, T. Taniguchi, R. J. Cava, P. Jarillo-Herrero, Electrically tunable low-density superconductivity in a monolayer topological insulator. *Science* **362**, 926–929 (2018).

20. Y. Yu, L. Ma, P. Cai, R. Zhong, C. Ye, J. Shen, G. D. Gu, X. H. Chen, Y. Zhang, High-temperature superconductivity in monolayer $Bi_2Sr_2CaCu_2O_{8+\delta}$. *Nature* **575**, 156–163 (2019).

21. T. Zhang, P. Cheng, W.-J. Li, Y.-J. Sun, G. Wang, X.-G. Zhu, K. He, L. Wang, X. Ma, X. Chen, Y. Wang, Y. Liu, H.-Q. Lin, J.-F. Jia, Q.-K. Xue, Superconductivity in one-atomic-layer metal films grown on Si(111). *Nat. Phys.* **6**, 104–108 (2010).

22. N. Reyren, S. Thiel, A. D. Caviglia, L. F. Kourkoutis, G. Hammerl, C. Richter, C. W. Schneider, T. Kopp, A. S. Ruetschi, D. Jaccard, M. Gabay, D. A. Muller, J. M. Triscone, J. Mannhart, Superconducting interfaces between insulating oxides. *Science* **317**, 1196–1199 (2007).

23. Z. Chen, Y. Liu, H. Zhang, Z. R. Liu, H. Tian, Y. Q. Sun, M. Zhang, Y. Zhou, J. R. Sun, Y. W. Xie, Electric field control of superconductivity at the $LaAlO_3/KTaO_3$(111) interface. *Science* **372**, 721–724 (2021).

24. G. Herranz, G. Singh, N. Bergeal, A. Jouan, J. Lesueur, J. Gazquez, M. Varela, M. Scigaj, N. Dix, F. Sanchez, J. Fontcuberta, Engineering two-dimensional superconductivity and Rashba spin-orbit coupling in $LaAlO_3/SrTiO_3$ quantum wells by selective orbital occupancy. *Nat. Commun.* **6**, 6028 (2015).

25. Y. Xing, H. M. Zhang, H. L. Fu, H. Liu, Y. Sun, J. P. Peng, F. Wang, X. Lin, X. C. Ma, Q. K. Xue, J. Wang, X. C. Xie, Quantum Griffiths singularity of superconductor-metal transition in Ga thin films. *Science* **350**, 542–545 (2015).

26. S. Y. Qin, J. Kim, Q. Niu, C. K. Shih, Superconductivity at the two-dimensional limit. *Science* **324**, 1314–1317 (2009).

27. I. Bozovic, X. He, J. Wu, A. T. Bollinger, Dependence of the critical temperature in overdoped copper oxides on superfluid density. *Nature* **536**, 309–311 (2016).

28. Y. Mizukami, H. Shishido, T. Shibauchi, M. Shimozawa, S. Yasumoto, D. Watanabe, M. Yamashita, H. Ikeda, T. Terashima, H. Kontani, Y. Matsuda, Extremely strong-coupling superconductivity in artificial two-dimensional Kondo lattices. *Nat. Phys.* **7**, 849–853 (2011).

29. A. Devarakonda, H. Inoue, S. Fang, C. Ozsoy-Keskinbora, T. Suzuki, M. Kriener, L. Fu, E. Kaxiras, D. C. Bell, J. G. Checkelsky, Clean 2D superconductivity in a bulk van der Waals superlattice. *Science* **370**, 231–236 (2020).

30. Y. Saito, Y. Kasahara, J. Ye, Y. Iwasa, T. Nojima, Metallic ground state in an ion-gated two-dimensional superconductor. *Science* **350**, 409–413 (2015).

31. X. Li, J. Sun, P. Shahi, M. Gao, A. H. MacDonald, Y. Uwatoko, T. Xiang, J. B. Goodenough, J. Cheng, J. Zhou, Pressure-induced phase transitions and superconductivity in a black phosphorus single crystal. *Proc. Natl. Acad. Sci. U.S.A.* **115**, 9935–9940 (2018).

32. J. C. Jamieson, Crystal structures adopted by black phosphorus at high pressures. *Science* **139**, 1291–1292 (1963).

33. I. Shirotani, S. Shiba, K. Takemura, O. Shimomura, T. yagi, Pressure-induced phase transitions of phosphorus-arsenic alloys. *Phys. B Condens. Matter* **190**, 169–176 (1993).

34. J. Guo, H. Wang, F. von Rohr, W. Yi, Y. Zhou, Z. Wang, S. Cai, S. Zhang, X. Li, Y. Li, J. Liu, K. Yang, A. Li, S. Jiang, Q. Wu, T. Xiang, R. J. Cava, L. Sun, Electron-hole balance and the anomalous pressure-dependent superconductivity in black phosphorus. *Phys. Rev. B* **96**, 224513 (2017).

35. V. G. Kogan, R. Prozorov, Interband coupling and nonmagnetic interband scattering in ±*s* superconductors. *Phys. Rev. B* **93**, 224515 (2016).

36. M. Liao, Y. Zang, Z. Guan, H. Li, Y. Gong, K. Zhu, X.-P. Hu, D. Zhang, Y. Xu, Y.-Y. Wang, K. He, X.-C. Ma, S.-C. Zhang, Q.-K. Xue, Superconductivity in few-layer stanene. *Nat. Phys.* **14**, 344–348 (2018).

37. B. Y. Wang, D. Li, B. H. Goodge, K. Lee, M. Osada, S. P. Harvey, L. F. Kourkoutis, M. R. Beasley, H. Y. Hwang, Isotropic Pauli-limited superconductivity in the infinite-layer nickelate $Nd_{0.775}Sr_{0.225}NiO_2$. *Nat. Phys.* **17**, 473–477 (2021).

38. J. Guo, Y. Zhou, C. Huang, S. Cai, Y. Sheng, G. Gu, C. Yang, G. Lin, K. Yang, A. Li, Q. Wu, T. Xiang, L. Sun, Crossover from two-dimensional to three-dimensional superconducting states in bismuth-based cuprate superconductor. *Nat. Phys.* **16**, 295–300 (2019).

39. J. Bardeen, L. N. Cooper, J. R. Schrieffer, Theory of superconductivity. *Phys. Rev.* **108**, 1175–1204 (1957).

40. X. Bi, Z. Li, J. Huang, F. Qin, C. Zhang, Z. Xu, L. Zhou, M. Tang, C. Qiu, P. Tang, T. Ideue, T. Nojima, Y. Iwasa, H. Yuan, Orbital-selective two-dimensional superconductivity in 2H-NbS_2. *Phys. Rev. Res.* **4**, 013188 (2022).

41. J. W. Hsu, A. Kapitulnik, A. Superconducting transition, fluctuation, and vortex motion in a two-dimensional single-crystal Nb film. *Phys. Rev. B* **45**, 4819–4835 (1992).

42. D. A. Dikin, M. Mehta, C. W. Bark, C. M. Folkman, C. B. Eom, V. Chandrasekhar, Coexistence of superconductivity and ferromagnetism in two dimensions. *Phys. Rev. Lett.* **107**, 056802 (2011).

43. M. Ben Shalom, M. Sachs, D. Rakhmilevitch, A. Palevski, Y. Dagan, Tuning spin-orbit coupling and superconductivity at the $SrTiO_3$/$LaAlO_3$ interface: A magnetotransport study. *Phys. Rev. Lett.* **104**, 126802 (2010).

44. L. Chen, G. Zhou, Z. Liu, X. Ma, J. Chen, Z. Zhang, X. Ma, F. Li, H. M. Cheng, W. Ren, Scalable clean exfoliation of high-quality few-layer black phosphorus for a flexible lithium ion battery. *Adv. Mater.* **28**, 510–517 (2016).

45. A. D. Chijioke, W. J. Nellis, A. Soldatov, I. F. Silvera, The ruby pressure standard to 150GPa. *J. Appl. Phys.* **98**, 114905 (2005).

46. J. P. Perdew, A. Ruzsinszky, G. I. Csonka, O. A. Vydrov, G. E. Scuseria, L. A. Constantin, X. Zhou, K. Burke, Restoring the density-gradient expansion for exchange in solids and surfaces. *Phys. Rev. Lett.* **100**, 136406 (2008).

47. G. Kresse, J. Furthmuller, Efficient iterative schemes for ab initio total-energy calculations using a plane-wave basis set. *Phys. Rev. B* **54**, 11169–11186 (1996).

48. G. Kresse, D. Joubert, From ultrasoft pseudopotentials to the projector augmented-wave method. *Phys. Rev. B* **59**, 1758–1775 (1999).

49. F. D. Murnaghan, The compressibility of media under extreme pressures. *Proc. Natl. Acad. Sci. U.S.A.* **30**, 244–247 (1944).

50. F. Birch, Finite elastic strain of cubic crystals. *Phys. Rev.* **71**, 809–824 (1947).

51. F. Birch, Finite strain isotherm and velocities for single-crystal and polycrystalline NaCl at high pressures and 300°K. *J. Geophys. Res.* **83**, 1257–1268 (1978).

52. F. Xia, H. Wang, Y. Jia, Rediscovering black phosphorus as an anisotropic layered material for optoelectronics and electronics. *Nat. Commun.* **5**, 4458 (2014).

53. F. Sheng, C. Hua, M. Cheng, J. Hu, X. Sun, Q. Tao, H. Lu, Y. Lu, M. Zhong, K. Watanabe, T. Taniguchi, Q. Xia, Z. A. Xu, Y. Zheng, Rashba valleys and quantum Hall states in few-layer black arsenic. *Nature* **593**, 56–60 (2021).

54. A. Kundu, D. Tristant, N. Sheremetyeva, A. Yoshimura, A. Torres Dias, K. S. Hazra, V. Meunier, P. Puech, Reversible pressure-induced partial phase transition in few-layer black phosphorus. *Nano Lett.* **20**, 5929–5935 (2020).

55. A. Brown, S. Rundqvist, Refinement of the crystal structure of black phosphorus. *Acta Crystallogr.* **19**, 684–685 (1965).

56. L. Cartz, S. R. Srinivasa, R. J. Riedner, J. D. Jorgensen, T. G. Worlton, Effect of pressure on bonding in black phosphorus. *J. Chem. Phys.* **71**, 1718–1721 (1979).

57. N. R. Werthamer, E. Helfand, P. C. Hohenberg, Temperature and purity dependence of the superconducting critical field, H_{c2}. III. Electron spin and spin-orbit effects. *Phys. Rev.* **147**, 295–302 (1966).

58. S. K. Goh, Y. Mizukami, H. Shishido, D. Watanabe, S. Yasumoto, M. Shimozawa, M. Yamashita, T. Terashima, Y. Yanase, T. Shibauchi, A. I. Buzdin, Y. Matsuda, Anomalous upper critical field in $CeCoIn_5/YbCoIn_5$ superlattices with a Rashba-type heavy Fermion interface. *Phys. Rev. Lett.* **109**, 157006 (2012).

59. M. R. Beasley, J. E. Mooij, T. P. Orlando, Possibility of vortex-antivortex pair dissociation in two-dimensional superconductors. *Phys. Rev. Lett.* **42**, 1165–1168 (1979).

60. B. I. Halperin, D. R. Nelson, Resistive transition in superconducting films. *J. Low Temp. Phys.* **36**, 599–616 (1979).

61. Y. H. Lin, J. Nelson, A. M. Goldman, Suppression of the Berezinskii-Kosterlitz-Thouless transition in 2D superconductors by macroscopic quantum tunneling. *Phys. Rev. Lett.* **109**, 017002 (2012).

62. P. G. Baity, X. Shi, Z. Shi, L. Benfatto, D. Popović, Effective two-dimensional thickness for the Berezinskii-Kosterlitz-Thouless-like transition in a highly underdoped $La_{2-x}Sr_xCuO_4$. *Phys. Rev. B* **93**, 024519 (2016).

63. J. Pearl, Current distribution in superconducting films carrying quantized fluxoids. *Appl. Phys. Lett.* **5**, 65–66 (1964).

64. S. C. de la Barrera, M. R. Sinko, D. P. Gopalan, N. Sivadas, K. L. Seyler, K. Watanabe, T. Taniguchi, A. W. Tsen, X. Xu, D. Xiao, B. M. Hunt, Tuning Ising superconductivity with layer and spin-orbit coupling in two-dimensional transition-metal dichalcogenides. *Nat. Commun.* **9**, 1427 (2018).

65. A. Nader, P. Monceau, Critical field of 2H-$NbSe_2$ down to 50 mK. *Springerplus* **3**, 16 (2014).

66. J. Cui, P. Li, J. Zhou, W. Y. He, X. Huang, J. Yi, J. Fan, Z. Ji, X. Jing, F. Qu, Z. G. Cheng, C. Yang, L. Lu, K. Suenaga, J. Liu, K. T. Law, J. Lin, Z. Liu, G. Liu, Transport evidence of asymmetric spin-orbit coupling in few-layer superconducting 1Td-$MoTe_2$. *Nat. Commun.* **10**, 2044 (2019).

67. C. Liu, C.-S. Lian, M.-H. Liao, Y. Wang, Y. Zhong, C. Ding, W. Li, C.-L. Song, K. He, X.-C. Ma, W. Duan, D. Zhang, Y. Xu, L. Wang, Q.-K. Xue, Two-dimensional superconductivity and topological states in $PdTe_2$ thin films. *Phys. Rev. Mater.* **2**, 094001 (2018).

68. C. H. Sharma, A. P. Surendran, S. S. Varma, M. Thalakulam, 2D superconductivity and vortex dynamics in 1T-MoS_2. *Commun. Phys.* **1**, 90 (2018).

69. A. Yazdani, A. Kapitulnik, Superconducting-insulating transition in two-dimensional a-MoGe thin films. *Phys. Rev. Lett.* **74**, 3037–3040 (1995).

70. M. Yamada, T. Hirahara, S. Hasegawa, Magnetoresistance measurements of a superconducting surface state of in-induced and Pb-induced structures on Si(111). *Phys. Rev. Lett.* **110**, 237001 (2013).

71. M. Nazir, X. Yang, H. Tian, P. Song, Z. Wang, Z. Xiang, X. Guo, Y. Jin, L. You, D. Zheng, Investigation of dimensionality in superconducting NbN thin film samples with different thicknesses and NbTiN meander nanowire samples by measuring the upper critical field. *Chin. Phys. B* **29**, 087401 (2020).

72. N. Pinto, S. J. Rezvani, A. Perali, L. Flammia, M. V. Milosevic, M. Fretto, C. Cassiago, N. De Leo, Dimensional crossover and incipient quantum size effects in superconducting niobium nanofilms. *Sci. Rep.* **8**, 4710 (2018).

73. P. Spathis, H. Aubin, A. Pourret, K. Behnia, Nernst effect in the phase-fluctuating superconductor InO_x. *EPL* **83**, 57005 (2008).

74. Z. Chen, Z. Liu, Y. Sun, X. Chen, Y. Liu, H. Zhang, H. Li, M. Zhang, S. Hong, T. Ren, C. Zhang, H. Tian, Y. Zhou, J. Sun, Y. Xie, Two-dimensional superconductivity at the $LaAlO_3$/$KTaO_3$(110) heterointerface. *Phys. Rev. Lett.* **126**, 026802 (2021).

75. C. Liu, X. Yan, D. Jin, Y. Ma, H. W. Hsiao, Y. Lin, T. M. Bretz-Sullivan, X. Zhou, J. Pearson, B. Fisher, J. S. Jiang, W. Han, J. M. Zuo, J. Wen, D. D. Fong, J. Sun, H. Zhou, A. Bhattacharya, Two-dimensional superconductivity and anisotropic transport at $KTaO_3$ (111) interfaces. *Science* **371**, 716–721 (2021).

76. Y. Guo, J. Peng, W. Qin, J. Zeng, J. Zhao, J. Wu, W. Chu, L. Wang, C. Wu, Y. Xie, Freestanding cubic ZrN single-crystalline films with two-dimensional superconductivity. *J. Am. Chem. Soc.* **141**, 10183–10187 (2019).

77. Q. L. He, H. Liu, M. He, Y. H. Lai, H. He, G. Wang, K. T. Law, R. Lortz, J. Wang, I. K. Sou, Two-dimensional superconductivity at the interface of a Bi_2Te_3/FeTe heterostructure. *Nat. Commun.* **5**, 4247 (2014).

78. Q. Fan, W. H. Zhang, X. Liu, Y. J. Yan, M. Q. Ren, R. Peng, H. C. Xu, B. P. Xie, J. P. Hu, T. Zhang, D. L. Feng, Plain s-wave superconductivity in single-layer FeSe on $SrTiO_3$ probed by scanning tunnelling microscopy. *Nat. Phys.* **11**, 946–952 (2015).

79. W.-H. Zhang, Y. Sun, J.-S. Zhang, F.-S. Li, M.-H. Guo, Y.-F. Zhao, H.-M. Zhang, J.-P. Peng, Y. Xing, H.-C. Wang, T. Fujita, A. Hirata, Z. Li, H. Ding, C.-J. Tang, M. Wang, Q.-Y. Wang, K. He, S.-H. Ji, X. Chen, J.-F. Wang, Z.-C. Xia, L. Li, Y.-Y. Wang, J. Wang, L.-L. Wang, M.-W. Chen, Q.-K. Xue, X.-C. Ma, Direct observation of high-temperature superconductivity in one-unit-cell fese films. *Chin. Phys. Lett.* **31**, 017401 (2014).

80. J. Shiogai, Y. Ito, T. Mitsuhashi, T. Nojima, A. Tsukazaki, Electric-field-induced superconductivity in electrochemically etched ultrathin FeSe films on $SrTiO_3$ and MgO. *Nat. Phys.* **12**, 42–46 (2015).

81. J. Liang, Y. J. Zhang, X. Yao, H. Li, Z. X. Li, J. Wang, Y. Chen, I. K. Sou, Studies on the origin of the interfacial superconductivity of $Sb_2Te_3/Fe_{1+y}Te$ heterostructures. *Proc. Natl. Acad. Sci. U.S.A.* **117**, 221–227 (2020).

82. H. Yang, G. Chen, X. Zhu, J. Xing, H.-H. Wen, BCS-like critical fluctuations with limited overlap of Cooper pairs in FeSe. *Phys. Rev. B* **96**, 064501 (2017).

83. C. K. H. Borg, X. Zhou, C. Eckberg, D. J. Campbell, S. R. Saha, J. Paglione, E. E. Rodriguez, Strong anisotropy in nearly ideal tetrahedral superconducting FeS single crystals. *Phys. Rev. B* **93**, 094522 (2016).

84. J. Hanisch, K. Iida, F. Kurth, E. Reich, C. Tarantini, J. Jaroszynski, T. Forster, G. Fuchs, R. Huhne, V. Grinenko, L. Schultz, B. Holzapfel, High field superconducting properties of $Ba(Fe_{1-x}Co_x)_2As_2$ thin films. *Sci. Rep.* **5**, 17363 (2015).

85. S. V. Chong, S. Hashimoto, K. Kadowaki, Upper critical fields and critical current density of single crystal. *Solid State Commun.* **150**, 1178–1181 (2010).

86. E. D. Mun, M. M. Altarawneh, C. H. Mielke, V. S. Zapf, R. Hu, S. L. Bud'ko, P. C. Canfield, Anisotropic H_{c2} of $K_{0.8}Fe_{1.76}Se_2$ determined up to 60 T. *Phys. Rev. B* **83**, 100514 (2011).

87. J. L. Zhang, L. Jiao, F. F. Balakirev, X. C. Wang, C. Q. Jin, H. Q. Yuan, Upper critical field and its anisotropy in LiFeAs. *Phys. Rev. B* **83**, 174506 (2011).

88. H. Lei, R. Hu, E. S. Choi, J. B. Warren, C. Petrovic, Pauli-limited upper critical field of $Fe_{1+y}Te_{1-x}$ Se_x. *Phys. Rev. B* **81**, 094518 (2010).

89. A. P. Mackenzie, R. K. W. Haselwimmer, A. W. Tyler, G. G. Lonzarich, Y. Mori, S. Nishizaki, Y. Maeno, Extremely strong dependence of superconductivity on disorder in Sr_2RuO_4. *Phys. Rev. Lett.* **80**, 3890 (1998).

90. S. Foner, E. J. McNiff Jr., High-field measurements of anisotropy of H_{c2} and effect on grain-boundary flux pinning in V_3Si. *Appl. Phys. Lett.* **32**, 122–123 (1978).

91. Y. Qi, W. Shi, P. Werner, P. G. Naumov, W. Schnelle, L. Wang, K. G. Rana, S. Parkin, S. A. Medvedev, B. Yan, C. Felser, Pressure-induced superconductivity and topological quantum phase transitions in a quasi-one-dimensional topological insulator: Bi_4I_4. *npj Quant. Mater.* **3**, 4 (2018).

92. E. Cheng, W. Xia, X. Shi, Z. Yu, L. Wang, L. Yan, D. C. Peets, C. Zhu, H. Su, Y. Zhang, D. Dai, X. Wang, Z. Zou, N. Yu, X. Kou, W. Yang, W. Zhao, Y. Guo, S. Li, Pressure-induced

superconductivity and topological phase transitions in the topological nodal-line semimetal $SrAs_3$. *npj Quant. Mater.* **5**, 38 (2020).

93. W. Wu, J. Cheng, K. Matsubayashi, P. Kong, F. Lin, C. Jin, N. Wang, Y. Uwatoko, J. Luo, Superconductivity in the vicinity of antiferromagnetic order in CrAs. *Nat. Commun.* **5**, 5508 (2014).

94. K. Shimizu, H. Ishikawa, D. Takao, T. Yagi, K. Amaya, Superconductivity in compressed lithium at 20 K. *Nature* **419**, 597–599 (2002).

95. N. Tateiwa, T. C. Kobayashi, K. Hanazono, K. Amaya, Y. Haga, R. Settai, Y. Onuki, Pressure-induced superconductivity in a ferromagnet UGe_2. *J. Phys. Condens. Matter* **13**, L17–L23 (2001).

96. F. H. Yu, D. H. Ma, W. Z. Zhuo, S. Q. Liu, X. K. Wen, B. Lei, J. J. Ying, X. H. Chen, Unusual competition of superconductivity and charge-density-wave state in a compressed topological kagome metal. *Nat. Commun.* **12**, 3645 (2021).
